# HPV E6/E7‐Induced Acetylation of a Peptide Encoded by a Long Non‐Coding RNA Inhibits Ferroptosis to Promote the Malignancy of Cervical Cancer

**DOI:** 10.1002/advs.202414018

**Published:** 2025-01-21

**Authors:** Xiaoyu Qi, Jing Zhou, Xinyue Wang, Yan Shen, Yuxun Cao, Liangzi Jiang, Miaomiao Shen, Haoran Zhang, Tianjiao Wang, Pengjun Wei, Ruoqi Xu, Yue Yang, Xiangya Ding, Cong Wang, Xuemei Jia, Qin Yan, Wan Li, Chun Lu

**Affiliations:** ^1^ Department of Gynecology Women's Hospital of Nanjing Medical University Nanjing Women and Children's Healthcare Hospital Nanjing Medical University Nanjing 210004 P. R. China; ^2^ Department of Microbiology Nanjing Medical University Nanjing 211166 P. R. China; ^3^ Department of Pathology the First Affiliated Hospital of Nanjing Medical University Nanjing Medical University Nanjing 210029 P. R. China; ^4^ Key Laboratory of Pathogen Biology of Jiangsu Province Nanjing Medical University Nanjing 211166 P. R. China; ^5^ Department of Infectious Diseases Changzhou Third People's Hospital Changzhou Medical Center Nanjing Medical University Nanjing 211166 P. R. China

**Keywords:** acetylation, cervical cancer, ferroptosis, HPV, TUBORF, tumorigenesis

## Abstract

Although a fraction of functional peptides concealed within long non‐coding RNAs (lncRNAs) is identified, it remains unclear whether lncRNA‐encoded peptides are involved in the malignancy of cervical cancer (CC). Here, a 92‐amino acid peptide is discovered, which is named TUBORF, encoded by lncRNA TUBA3FP and highly expressed in CC tissues. TUBORF inhibits ferroptosis to promote the malignant proliferation of CC cells. Mechanistically, human papillomavirus (HPV) oncogenes E6 and E7 upregulate TUBORF through CREB‐binding protein (CBP)/E1A‐binding protein p300 (p300)‐mediated histone H3 lysine 27 acetylation (H3K27ac) of lncTUBA3FP enhancer. Furthermore, E6 and E7 elevate and recruit acetyltransferase establishment of sister chromatid cohesion N‐acetyltransferase 1 (ESCO1) to bind to and acetylate TUBORF, which facilitates the degradation of immunity‐related GTPase Q (IRGQ) via a ubiquitin‐proteasome pathway, resulting in the inhibition of ferroptosis and promotion of the malignant proliferation of CC cells. Importantly, silencing ESCO1 or TURORF amplifies anticancer effects by paclitaxel both in CC cells and in vivo. These novel findings reveal oncopeptide TUBORF and its acetyltransferase ESCO1 as important regulators of ferroptosis and tumorigenesis during cervical cancer pathogenesis and establish the scientific basis for targeting these molecules for treating CC.

## Graphical abstract

Qi et al. report that TUBORF, a peptide encoded by lncTUBA3FP, inhibits ferroptosis to promote the malignancy of cervical cancer. HPV oncoproteins E6 and E7 upregulate TUBORF expression through CBP/p300‐mediated H3K27 acetylation of lncTUBA3FP enhancer. E6 and E7 also elevate and recruit acetyltransferase ESCO1 to bind to and acetylate TUBORF, which facilitates the degradation of IRGQ via a ubiquitin‐proteasome pathway.

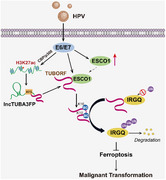

## 1 Introduction

Early screening and human papillomavirus (HPV) vaccination have reduced the mortality rate of cervical cancer (CC),^[^ ^1^ ^]^ but it is still the second most prevalent cancer in low‐ and middle‐income countries and the fourth worldwide.^[^ ^2^ ^]^ The World Health Organization estimated 604 000 new cases of CC and 342 000 related deaths in 2021.^[^ ^2a^ ^]^ The main risk factors of CC include high‐risk HPV (hrHPV) infection, age, smoking, delivery, use of oral contraceptives, and diet.^[^ ^3^ ^]^ Among these, persistent hrHPV infection is the culprit.^[^ ^3^ ^]^ HPV 16 and 18 infections account for 50% and 10% of CC cases, respectively.^[^ ^4^ ^]^ The virulence of HPV is bequeathed by HPV oncoproteins E6 and E7, which activate the proliferation and inhibit the apoptosis of cancer cells, and help them evade immune traps.^[^ ^5^ ^]^ Free from E6 and E7, CC cells will undergo senescence or apoptosis.^[^ ^5b^ ^]^ CC is commonly managed through standard treatment modalities including chemotherapy, surgery, and radiation therapy. Chemotherapy plays a vital role in CC treatment, with drugs such as paclitaxel, cisplatin, 5‐fluorouracil, and leucovorin. Paclitaxel has emerged as the global standard cytotoxic agent for patients with metastatic or recurrent CC.^[^ ^6^ ^]^ However, the development of chemoresistance due to the upregulation of relevant drug transporters remains a major obstacle to successful treatment outcomes. Combining multiple drugs has been proposed as a strategy to counter drug resistance. Nonetheless, chemotherapy drugs are associated with significant drawbacks including adverse effects such as kidney disease, nausea/vomiting, and neurotoxicity. Addressing these limitations necessitates the discovery of new chemotherapeutic targets to improve the efficacy and safety profiles of CC treatment regimens.

Long non‐coding RNAs (lncRNAs) are initially known as non‐protein coding transcripts. However, ribosome profiling sequencing (Ribo‐seq) and mass spectrometry have unveiled that the open reading frames (ORFs) of some lncRNAs can encode functional peptides.^[^ ^7^ ^]^ The roles of lncRNA‐encoded peptides in a variety of diseases, such as muscle physiology and immune, have been substantiated. For instance, a conserved micro peptide MLN encoded by an lncRNA regulates muscle performance.^[^ ^8^ ^]^ Mitochondrial micro peptide 47 controls immune response by activating NOD‐like receptor family pyrin domain containing (NLRP3) inflammasome to monitor pathogens.^[^ ^9^ ^]^ The small lncRNA peptides are also related to cancer progression. For example, lncRNA peptide HOXB‐AS3 suppresses colon cancer growth.^[^ ^7a^ ^]^ Peptide SMIM30 encoded by LINC00998 potentiates cell proliferation and migration to promote hepatocellular carcinoma.^[^ ^7b^ ^]^ LncRNA‐encoded small protein SRSP propels the progression of colorectal cancer by regulating mRNA splicing.^[^ ^10^ ^]^ However, whether there are lncRNA‐coding peptides playing a role in CC pathogenesis remains unknown.

Ferroptosis is a newly discovered oxidation‐regulated cell death reined by iron‐dependent lipid peroxidation.^[^ ^11^ ^]^ Excessive free irons in cells produce hydroxyl radicals and highly reactive oxygen species (ROS) that can damage the structure and function of cells.^[^ ^12^ ^]^ Cells undergoing ferroptosis demonstrate dramatic mitochondrial fragmentation and cristae enlargement.^[^ ^13^ ^]^ The hallmarks of ferroptosis include i) iron overload on cells, ii) L‐ROS (lipid reactive oxygen species) accumulation, and iii) destruction of the glutathione (GSH)‐glutathione peroxidase 4 (GPX4) peroxide defense system. Fe^2+^ in cells can capture and transfer electrons to O_2_ and H_2_O_2_ to form harmful peroxides.^[^ ^14^ ^]^ Lipid peroxidation ensuing the Fenton reaction of redox‐active Fe^2+^ and H_2_O_2_ can produce a lot of ROS.^[^ ^14^ ^]^ Moreover, the defense system in cells against peroxidation benefits the elimination of L‐ROS. System Xc‐, a disulfide‐linked heterodimer composed of subunit solute carrier family members (SLC7A11 and SLC3A2),^[^ ^15^ ^]^ mediates the communication between extracellular cystine and intracellular glutamate, which is a key step in GSH synthesis. GSH is a cofactor of GPX4,^[^ ^16^ ^]^ and GPX4 can convert lipid hydroperoxides to lipid alcohols, thus preventing Fe^2+^‐dependent lipid ROS generation and ultimately inhibiting ferroptosis.^[^ ^17^ ^]^ Ferroptosis impedes tumor development by means of several tumor suppressors including p53 and BRCA1 associated deubiquitinase 1.^[^ ^18^ ^]^ Cancer cells, due to their active metabolism or higher ROS content, are more sensitive to ferroptosis.^[^ ^19^ ^]^ The tumor‐suppressive role of ferroptosis has been proven in breast cancer, pancreatic cancer, and ovarian cancer.^[^ ^20^, ^21^ ^]^ Previous studies have hinted that ferroptosis may be a mechanism fighting against CC;^[^ ^22^ ^]^ however, the detailed molecular mechanism remains to be elucidated.

The immunity‐related GTPase (IRG) family is ubiquitously expressed in both humans and mice, with the functional IRG‐type guanine nucleotide‐binding (G) domain.^[^ ^23^ ^]^ IRG belongs to the interferon regulatory protein family and plays a vital role in fighting against bacterial infections, inflammatory bowel disease, autoimmune diseases, and autophagy. This understanding contributes to a better comprehension of the pathogenesis of systemic diseases and tumors.^[^ ^24^ ^]^ As a member of the IRG family, immunity‐related GTPase Q (IRGQ) possesses a conserved G‐IRG domain and was initially found to be highly expressed in hepatocellular carcinoma tissues.^[^ ^25^ ^]^ However, the role of IRGQ in cervical cancer and ferroptosis is still unclear.

Establishment of sister chromatid cohesion N‐acetyltransferase 1 (ESCO1) is an acetyltransferase essential for the establishment of sister chromatid cohesion.^[^ ^26^, ^27^ ^]^ Sister chromatid cohesion is indispensable in mitosis, where it maintains genome integrity by ensuring accurate chromosome separation. Disruptions in this process can result in tumorigenesis or aneuploidy.^[^ ^28^ ^]^ Given that cohesion is also essential for chromosome recombination repair, impaired ESCO1 function leads to a significant increase in improper chromosome repair.^[^ ^29^ ^]^ Some evidence suggests that ESCO1 is involved in tumorigenesis.^[^ ^30^ ^]^ However, its role in cervical cancer remains unknown.

Here, we reported that HPV E6‐ and E7‐upregulated lncTUBA3FP could encode a 92‐amino acid peptide, which we named TUBA3FP open reading frame (TUBORF). TUBORF was highly expressed in CC tissues and HPV‐positive CC cells that inhibited ferroptosis to promote the malignant proliferation of CC cells. Mechanistically, E6 and E7 not only strengthened lncTUBA3FP level by CREB‐binding protein (CBP)/E1A binding protein p300 (p300)‐induced histone H3 lysine 27 acetylation (H3K27ac) of lncTUBA3FP enhancer but also upregulated and recruited acetyltransferase ESCO1 to bind to and acetylate TUBORF, which facilitated the degradation of IRGQ, resulting in inhibition of ferroptosis to promote the malignant proliferation of CC cells. Silencing ESCO1 or TUBORF amplified paclitaxel‐mediated anticancer effect both in CC cells and in vivo. Overall, our novel findings reveal that oncopeptide TUBORF and its acetyltransferase ESCO1 are important regulators of ferroptosis and tumorigenesis in CC cells, thereby identifying new therapeutic targets for CC.

## 2 Results

### 2.1 TUBORF, a Newly Discovered Peptide, is Encoded by lncRNA TUBA3FP and Highly Expressed in Cervical Cancer Tissues

**Figure 1:**
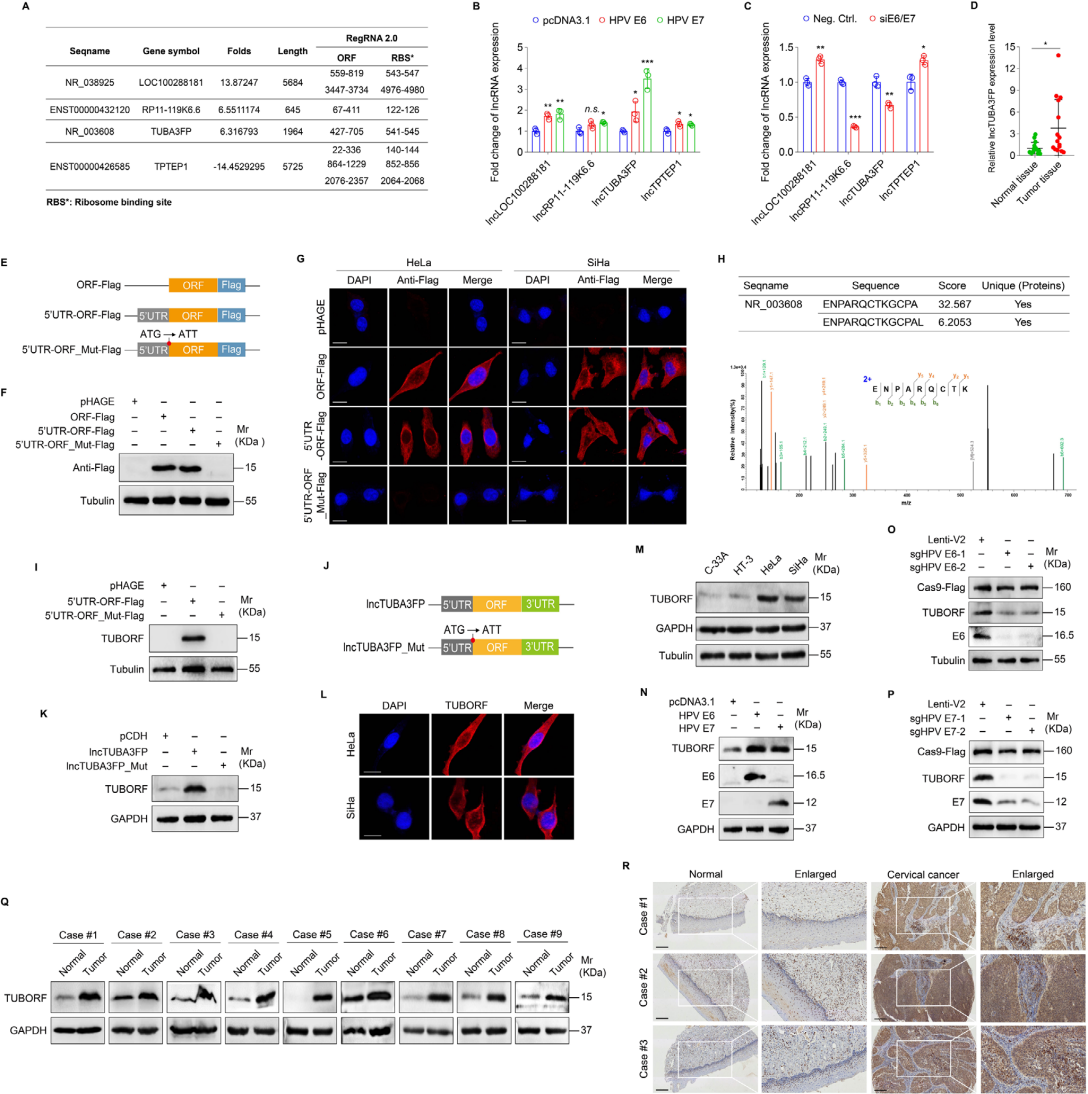
TUBORF is a naturally produced peptide and is highly expressed in cervical cancer. A) Differentially expressed lncRNAs between cervical cancer tissues and normal cervical epithelial tissues were analyzed by RegRNA2.0 software to predict their open reading frames (**ORF**) and ribosome binding sites (**RBS**). B) RT‐qPCR analysis of differentially expressed lncRNAs in (A) in HPV‐negative cells C‐33A transfected with HPV E6 and E7 constructs (**HPV E6, HPV E7**) or the control (**pcDNA3.1**) (*n* = 3). C) RT‐qPCR analysis of differentially expressed lncRNAs in (A) in HeLa cells transfected with an HPV E6/E7 siRNA pool (**siE6/E7**) or the control (**Neg. Ctrl**.) (*n* = 3). D) LncTUBA3FP level was detected by RT‐qPCR in cervical cancer tissues (**Tumor tissue**) and normal cervical epithelial tissues (**Normal tissue**) (*n* = 15). E) Pattern diagrams of ORF of TUBORF plus Flag (**ORF‐Flag**), 5′UTR plus ORF of TUBORF plus Flag (**5′UTR‐ORF‐Flag**) and 5′UTR plus mutant ORF of TUBORF plus Flag (**5′UTR‐ORF_Mut‐Flag**; the start codon ATG is mutated to ATT) constructs. F) Cervical cancer cells were transduced by 2 MOI lentiviral ORF of TUBORF plus Flag (**ORF‐Flag**), lentiviral 5′UTR plus ORF of TUBORF plus Flag (**5′UTR‐ORF‐Flag**), and lentiviral 5′UTR plus mutant ORF of TUBORF plus Flag (**5′UTR‐ORF_Mut‐Flag**; the start codon ATG is mutated to ATT) or the control (**pHAGE**). Western blotting analysis of TUBORF expression with anti‐Flag antibodies. G) HeLa and SiHa cells were transduced by 2 MOI lentiviral ORF of TUBORF plus Flag (**ORF‐Flag**), lentiviral 5′UTR plus ORF of TUBORF plus Flag (**5′UTR‐ORF‐Flag**) and lentiviral 5′UTR plus mutant ORF of TUBORF plus Flag (**5′UTR‐ORF_Mut‐Flag**; the start codon ATG is mutated to ATT) or the control (**pHAGE**). Immunofluorescence assay (**IFA**) showed co‐localization of TUBORF peptide with anti‐Flag antibodies. H) The peptide segments of TUBORF in HeLa cell lysate were repeatedly identified using mass spectrometry (**MS**). I) Cervical cancer cells were transduced by 2 MOI lentiviral 5′UTR plus ORF of TUBORF plus Flag (**5′UTR‐ORF‐Flag**) and lentiviral 5′UTR plus mutant ORF of TUBORF plus Flag (**5′UTR‐ORF_Mut‐Flag**; the start codon ATG is mutated to ATT) or the control (**pHAGE**). TUBORF peptide level was detected by Western blotting analysis with anti‐TUBORF antibodies. J) Diagram of lncTUBA3FP and lncTUBA3FP_Mut (the start codon ATG is mutated to ATT) constructs. K) Cervical cancer HeLa cells were transduced with 2 MOI lentiviral lncTUBA3FP (**lncTUBA3FP**), lncTUBA3FP_Mut (**lncTUBA3FP_Mut**) or the control (**pCDH**). The TUBORF peptide level was examined by Western blotting analysis with anti‐TUBORF antibodies. L) Immunofluorescence assay (**IFA**) for analysis of TUBORF peptide levels in HeLa and SiHa cells with anti‐TUBORF antibodies. M) Western blotting analysis of TUBORF peptide levels in HPV‐positive cells HeLa and SiHa, compared to those in HPV‐negative cells C‐33A and HT‐3. N) Western blotting analysis of TUBORF peptide levels in HPV‐negative cells C‐33A transfected with HPV E6 and E7 constructs (**HPV E6, HPV E7**) or the control (**pcDNA3.1**) with anti‐TUBORF antibodies. O) Western blotting analysis of TUBORF peptide levels in HeLa cells transduced with 2 MOI lentiviral sgRNAs of HPV E6 (**sgHPV E6‐1, sgHPV E6‐2**) or the control (**Lenti‐V2**) with anti‐TUBORF antibodies. P) Western blotting analysis of TUBORF peptide levels in HeLa cells transduced with 2 MOI lentiviral sgRNAs of HPV E7 (**sgHPV E7‐1, sgHPV E7‐2**) or the control (**Lenti‐V2**) with anti‐TUBORF antibodies. Q) Western blotting analysis of TUBORF peptide levels in the indicated cervical cancer tissues (**Tumor**) from 9 cases (**Case #1‐9**) and paired normal cervical epithelial tissues (**Normal**) (*n* = 9). R) Representative immunohistochemistry (IHC) staining images of TUBORF peptide expression in cervical cancer tissues (**Cervical cancer**) and their corresponding normal cervical epithelial tissues (**Normal**) from Case #1‐3. Scar bars, 100 µm.Data were presented with mean ± SD. ^*^ *p* < 0.05, ^**^ *p* < 0.01, and ^***^ *p* < 0.001, Student's *t*‐test. *n.s*., not significant.

To determine whether HPV oncoproteins E6 and E7 regulate lncRNAs with coding potential, we analyzed human lncRNA microarray data of CC tissues reported in our previous study,^[^ ^26^ ^]^ and predicted four potential coding lncRNAs using RegRNA2.0 software (**Figure** **1**A). With overexpression and knockdown of E6 and E7 in CC cells, respectively, we found that only lncTUBA3FP expression was regulated by HPV E6 and E7 (Figure 1B,C). We also analyzed the expression levels of lncTUBA3FP in both HPV‐negative cervical cancer cell lines (C‐33A and HT‐3) and HPV‐positive cervical cancer cell lines (HeLa and SiHa). We revealed that the expression level of lncTUBA3FP was significantly lower in C‐33A and HT‐3 compared to HeLa and SiHa (Figure S1A, Supporting Information). Similarly, we observed decreased expression levels of lncTUBA3FP in the HPV‐negative human tongue squamous cell lines SCC‐9 and CAL‐27, when compared to the HPV‐positive human tongue squamous cell lines UPCI‐SCC‐090 and UPCI‐SCC‐154 (Figure S1B, Supporting Information). We further examined the expression levels of lncTUBA3FP in 15 pairs of matched CC tissues and corresponding normal tissues. We showed that the expression level of lncTUBA3FP was upregulated in CC tissues (Figure 1D). LncTUBA3FP was predicted to have the potential to encode a 92‐amino acid peptide (Figure 1A), which we named TUBA3FP open reading frame (TUBORF). To validate whether TUBORF is encoded by lncTUBA3FP, we generated a series of constructs, in which the Flag tag was fused to the C terminus of the ORF or the 5′UTR‐ORF or the 5′UTR‐ORF_Mut (with a start codon ATG mutated to ATT) in the lncTUBA3FP transcript (Figure 1E). The expression of the lncTUBA3FP ORF‐Flag fusion protein was observed in ORF‐Flag‐ and 5′ UTR‐OFR‐Flag‐transduced cells, but not in 5′UTR‐ORF_Mut‐Flag‐transduced cells (Figure 1F). Similar results were also observed in CC cells with immunofluorescence staining assay (IFA) (Figure 1G). Importantly, a specific peptide encoded by the lncTUBA3FP ORF was identified in HeLa cell lysates by mass spectrometry (Figure 1H). These results suggest that the TUBORF peptide is naturally and endogenously expressed in CC cells.

Next, we produced an antibody against TUBORF to perform Western blotting for examination of the endogenous TUBORF in CC cells. We observed the expression of TUBORF in 5′UTR‐ORF‐Flag transduced cells, but not in 5′UTR‐ORF_Mut‐Flag‐transduced cells (Figure 1I), indicating that the anti‐TUBORF antibody was specific to TUBORF peptide. Furthermore, we showed that ATG mutation abolished the translation of TUBORF in lentiviral lncTUBA3FP_Mut (with the ATG‐mutated start codon)‐transduced cells (Figure 1J,K). IFA indicated that the TUBORF peptide was expressed in both the nucleus and cytoplasm of cervical cancer cells (Figure 1L). TUBORF expression level was higher in HPV‐positive cell lines than in HPV‐negative cells (Figure 1M; Figure S1C, Supporting Information), which was upregulated in HPV E6 and E7 overexpression C‐33A cells (Figure 1N), and decreased in HPV E6 or E7 knockdown HeLa cells (Figure 1O,P). Moreover, we detected TUBORF expression in nine pairs of matched CC tissues and corresponding normal tissues and found an upregulation of TUBORF expression in CC tissues (Figure 1Q). A tissue microarray analysis of 287 cases of cervical primary squamous cell carcinoma and 146 cases of normal squamous epithelium was performed using immunohistochemistry staining (IHC). Consistently, we showed a higher TUBORF expression level in CC tissues than in normal cervical epithelial tissues (Figure 1R). No significant correlation was found between TUBORF and various clinicopathological parameters in 287 cervical cancer patients, such as age, tumor size, depth of cervical stromal invasion, lymph node metastasis, lymphovascular invasion, histopathologic stage, and International Federation of Gynecology and Obstetrics (FIGO) stage (Table S1, Supporting Information), suggesting that TUBORF may primarily be implicated in the initiation of cervical cancer rather than its progression. Similarly, correlation analysis also showed no significant association between TUBORF and p16 as a surrogate marker of HPV infection status (Table S1, Supporting Information), which may be due to the high prevalence of p16 expression (98.6%) in the tissue microarray.

### 2.2 TUBORF Inhibits Ferroptosis and Promotes the Malignant Proliferation of Cervical Cancer Cells

**Figure 2:**
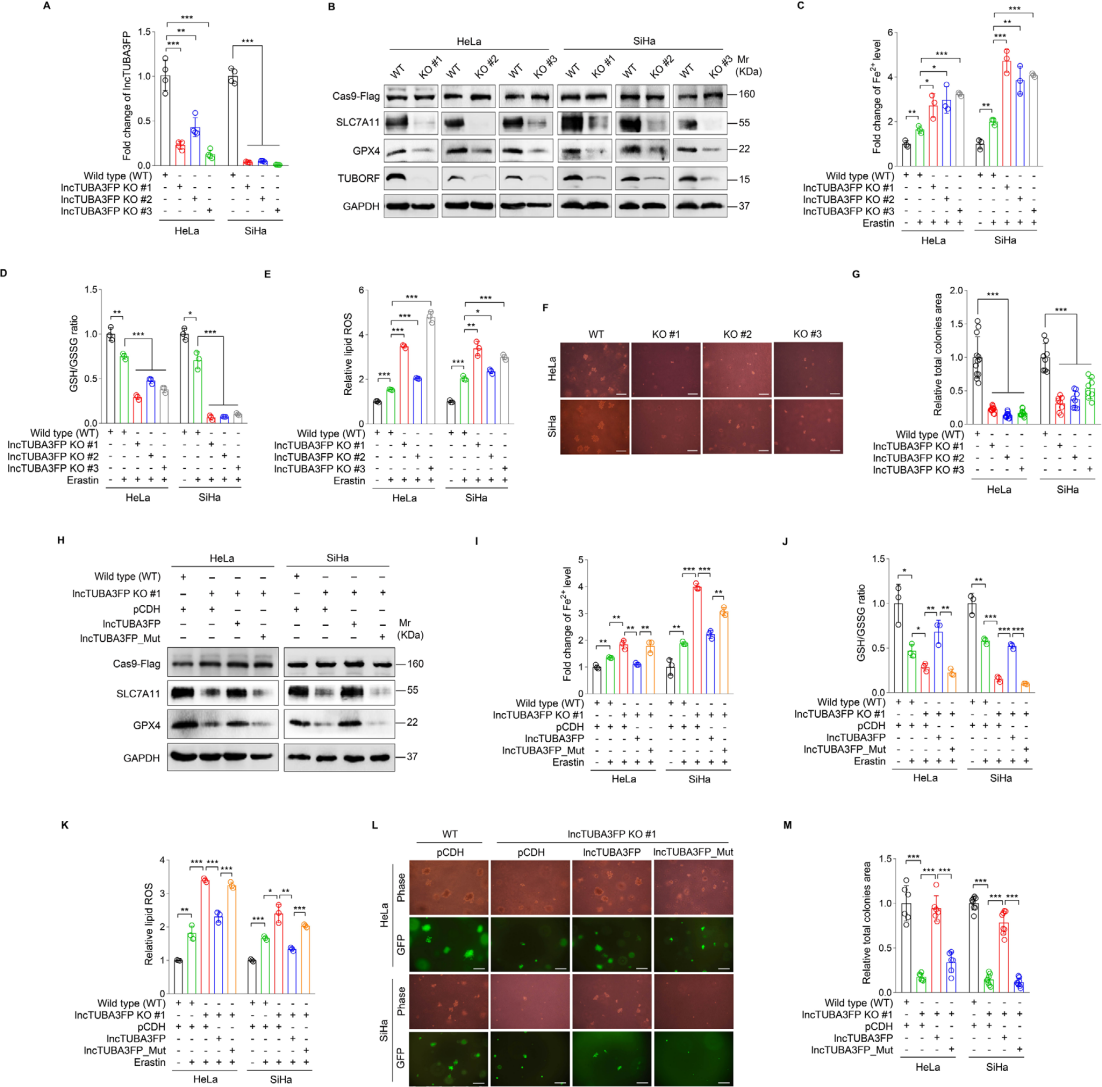
TUBORF peptide inhibits ferroptosis and promotes the malignant proliferation of cervical cancer cells. A) RT‐qPCR analysis of lncTUBA3FP level in lncTUBA3FP knockout HeLa and SiHa cells. Using the CRISPR/Cas9 method, three lncTUBA3FP knockout of cervical cancer monoclonal cell lines were screened and identified (**lncTUBA3FP KO #1, KO #2, and KO #3**) (*n* = 4). B) SCL7A11 and GPX4 protein levels in cells treated as in (A) and treated with 5 µm Erastin for 24 h were examined by Western blotting analysis. C) Cells treated as in (B) were employed to examine Fe^2+^ levels (*n* = 3). D) Cells treated as in (B) were used to measure GSH/GSSG levels (*n* = 3). E) Cells treated as in (B) were used to examine lipid ROS levels (*n* = 3). F) Soft agar colony formation assay was performed with cells treated as in (A). The representative images were photographed two weeks after cell seeding. Magnification, ×100. Scar bars, 40 µm. G) Results were quantified in (F) (*n* = 8‐12). H) Western blotting analysis of SLC7A11 and GPX4 expression in wild type (**WT**), or lncTUBA3FP knockout (**lncTUBA3FP KO #1**) cervical cancer cells infected with lentiviral lncTUBA3FP (**lncTUBA3FP**), lncTUBA3FP_Mut (**lncTUBA3FP_Mut**) or the control (**pCDH**), and treated with Erastin (5 µm, 24 h). I) Cells treated as in (H) were used to measure Fe^2+^ levels (*n* = 3). J) Cells treated as in (H) were employed to examine GSH/GSSG levels (*n* = 3). K) Cells treated as in (H) were used to measure lipid ROS levels (*n* = 3). L) Soft agar colony formation assay was performed with cells treated as in (H). The representative images were photographed two weeks after cell seeding. Magnification, ×100. Scar bars, 40 µm. M) Results were quantified in (L) (*n* = 6‐8). Data were presented with mean ± SD. ^*^ *p* < 0.05, ^**^ *p* < 0.01, and ^***^ *p* < 0.001, Student's *t*‐test.

Previous studies have demonstrated that ferroptosis suppresses various types of tumors; however, its mechanism in CC remains largely unknown.^[^ ^22^, ^27^ ^]^ To explore the influence of TUBORF on ferroptosis and tumorigenesis of CC cells, three lncTUBA3FP knockout (KO) monoclonal cell lines were established using CRISPR/Cas9 technique in both HeLa and SiHa cells (**Figure** **2**A). With the treatment of Erastin, a ferroptosis trigger, downregulation of SLC7A11 and GPX4 proteins was observed in lncTUBA3FP KO cells (Figure 2B). LncTUBA3FP knockout increased Erastin‐elicited intracellular Fe^2+^ levels (Figure 2C), reduced glutathione levels (Figure 2D), and upregulated lipid ROS levels (Figure 2E). Moreover, lncTUBA3FP knockout dramatically reduced the size of colonies of CC cells in soft agar assay (Figure 2F,G).

To exclude the possible effects of linear lncTUBA3FP, we overexpressed lncTUBA3FP or lncTUBA3FP_Mut in lncTUBA3FP KO cells. We found that lncTUBA3FP_Mut‐overexpressing cells were unable to achieve the same levels of ferroptosis and malignant proliferation as lncTUBA3FP‐overexpressing cells (Figure 2H–M). The exon region of the lncTUBA3FP transcript exhibits a 195 bp overlap with those of LRRC74B (Figure S2A, Supporting Information). A lower level of *LRRC74B* mRNA was detected in lncTUBA3FP KO cells (Figure S2B, Supporting Information). To exclude the influence of LRRC74B, we complemented LRRC74B in lncTUBA3FP KO cells and observed that complementing LRRC74B could not rescue the decreased malignant transformation ability of lncTUBA3FP KO cells (Figure S2C,D, Supporting Information). These data collectively suggest that TUBORF, rather than linear lncTUBA3FP or LRRC74B, inhibits ferroptosis and enhances the malignant proliferation of CC cells.

### 2.3 Histone Acetyltransferase CBP/p300 Promotes H3K27 Acetylation of lncTUBA3FP Enhancer to Upregulate Both lncTUBA3FP and TUBORF

**Figure 3:**
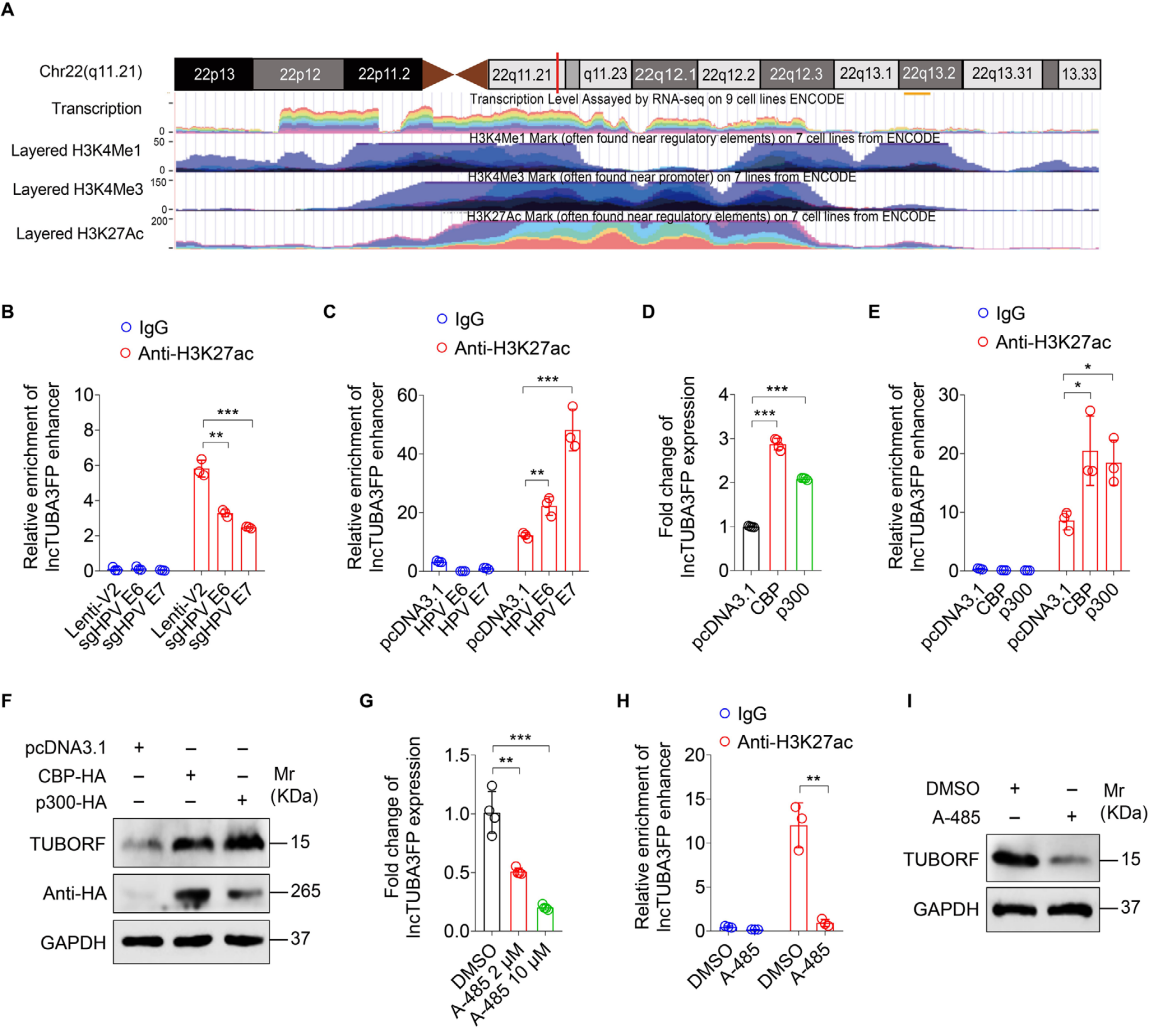
Histone acetyltransferase CBP/p300 promotes H3K27 acetylation of lncTUBA3FP enhancer. A) Predicted H3K4Me1, H3K4Me3, and H3K27ac enrichments at lncTUBA3FP enhancer region using the UCSC website. B) ChIP of the enrichment of H3K27ac in lncTUBA3FP enhancer region in E6 or E7 knockdown HeLa cells (**sgHPV E6, sgHPV E7**) or the control (**Lenti‐V2**) (*n* = 3). C) ChIP‐qPCR analysis of the H3K27ac level in lncTUBA3FP enhancer in C‐33A cells transfected with HPV E6 or E7 construct (**HPV E6, HPV E7**) or the control (**pcDNA3.1**) (*n* = 3). D) RT‐qPCR analysis of the lncTUBA3FP level in HeLa cells transfected with CBP‐HA (**CBP**), p300‐HA (**p300**), and their control (**pcDNA3.1**) (*n* = 3). E) H3K27ac level in lncTUBA3FP enhancer in HeLa cells treated as in (D) was detected by ChIP‐qPCR (*n* = 3). F) Western blotting analysis of the TUBORF peptide level in HeLa cells treated as in (D). G) HeLa cells were treated with A‐485 (2 or 10 µm in 24 h) or DMSO as the control. RT‐qPCR analysis was performed to examine the lncTUBA3FP level (*n* = 4). H) ChIP analysis of the H3K27ac level in lncTUBA3FP enhancer in HeLa cells treated with A‐485 (10 µm, 24 h) or DMSO as the control (*n* = 3). I) Western blotting analysis of the TUBORF peptide level in HeLa cells treated as in (H). Data were presented with mean ± SD. ^*^ *p* < 0.05, ^**^ *p* < 0.01, and ^***^ *p* < 0.001, Student's *t*‐test.

Previous studies have shown that lncRNA expression can be regulated by histone modification to cause transcriptional activation or inhibition.^[^ ^28^ ^]^ Using the UCSC database, we predicted that the enhancer regions of lncTUBA3FP were enriched with H3K4me1, H3K4me3, and H3K27ac (**Figure** **3**A). To validate this prediction, chromatin immunoprecipitation assay followed by RT‐qPCR (ChIP‐qPCR) was performed using anti‐H3K4me1, anti‐H3K4me3, and anti‐H3K27ac antibodies, respectively. We observed that H3K27ac and H3K4me1, but not H3K4me3, were enriched in lncTUBA3FP enhancer (Figure 3B; Figure S3, Supporting Information). Only a lower enrichment of H3K27ac was observed in both E6 and E7 knockdown cells (Figure 3B). Consistently, the enrichment of H3K27ac in lncTUBA3FP enhancer was higher in E6 and E7 overexpression cells (Figure 3C). Since H3K27ac modification is mediated by histone acetyltransferase CBP/p300 complex,^[^ ^29^ ^]^ we overexpressed CBP/p300 and observed a significant increase of lncTUBA3FP level in CC cells (Figure 3D). We further confirmed that the level of H3K27ac enriched in lncTUBA3FP enhancer was strikingly increased by CBP/p300 (Figure 3E). TUBORF expression was also upregulated by overexpression of CBP/p300 proteins (Figure 3F). Conversely, we treated CC cells with A‐485, a potent and selective catalytic inhibitor of CBP/p300, and observed that the level of lncTUBA3FP was downregulated in the treated cells in a dose‐dependent manner (Figure 3G). Importantly, treatment of CC cells with A‐485 not only inhibited the enrichment of H3K27ac in lncTUBA3FP enhancer but also repressed TUBORF peptide expression (Figure 3H,I). Together these data suggest that histone acetyltransferases CBP/p300 activate the H3K27 acetylation of lncTUBA3FP enhancer to upregulate both lncTUBA3FP and TUBORF.

### 2.4 TUBORF Acetylation Induced by Acetyltransferase ESCO1 Inhibits Ferroptosis and Promotes the Malignant Proliferation of Cervical Cancer Cells

**Figure 4:**
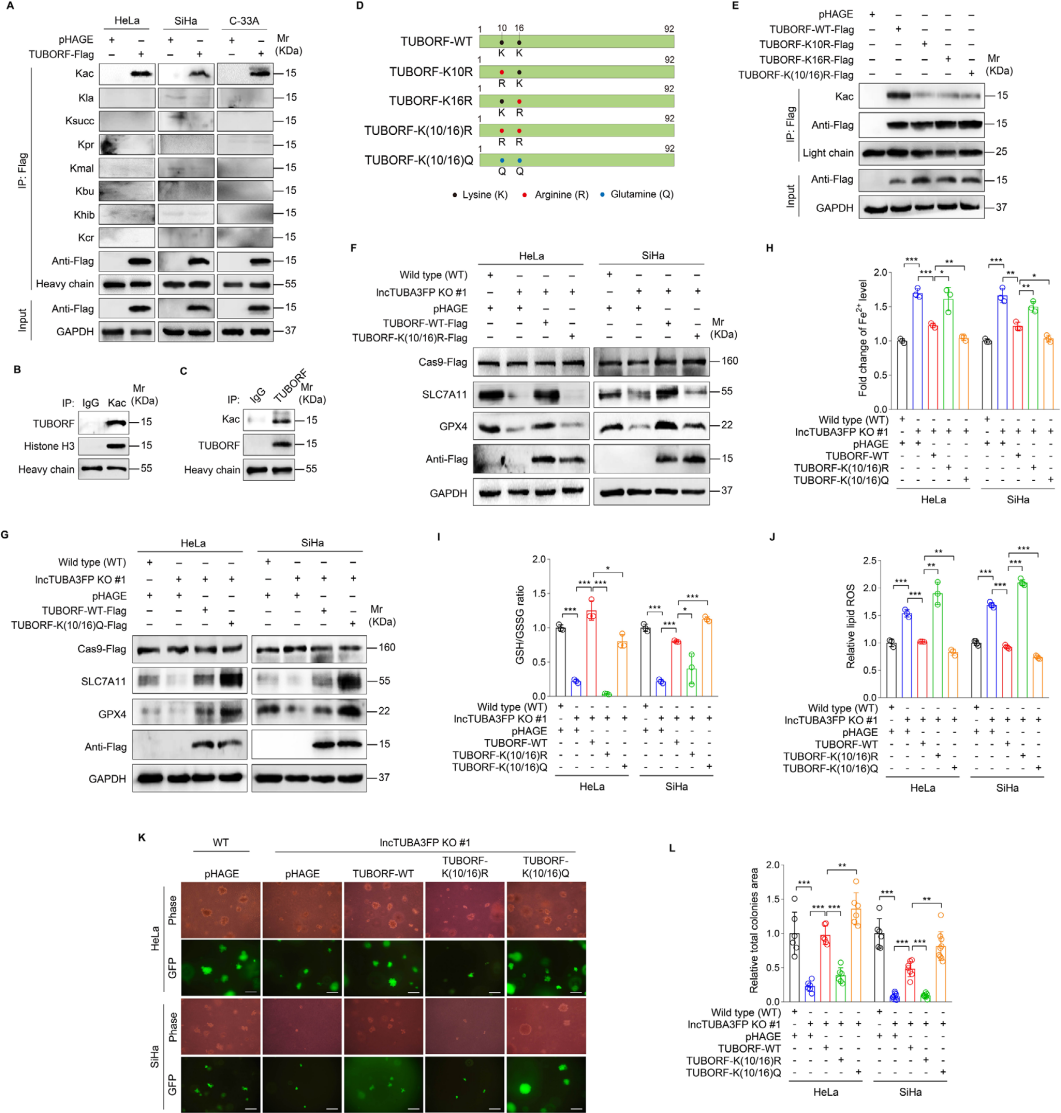
TUBORF acetylation suppresses ferroptosis and increases the malignant proliferation of cervical cancer cells. A) Cervical cancer cells transduced with 2 MOI lentiviral TUBORF (**TUBORF‐Flag**) or control (**pHAGE**). Cell proteins were pulled down by Flag antibodies and examined with pan‐antibodies specific to lysine acetylation (**Kac**), lactylation (**Kla**), succinylation (**Ksucc**), propionylation (**Kpr**), malonylation (**Kmal**), butyrylation (**Kbu**), hydroxyisobutyrylation (**Khib**), and crotonylation (**Kcr**), respectively. B) IP assay of endogenous acetylated TUBORF using the anti‐Kac antibody or IgG antibody as control in HeLa cells. C) IP assay of endogenous acetylated TUBORF using the anti‐TUBORF antibody or IgG antibody as a control in HeLa cells. D) Pattern diagram of constructs of wild type TUBORF (**TUBORF‐WT**), TUBORF‐K10R, TUBORF‐K16R, TUBORF‐K(10/16)R and TUBORF‐K(10/16)Q. E) IP assay was performed to examine the acetylation of TUBORF peptide in HeLa cells transduced with lentivirus TUBORF‐WT (**TUBORF‐WT**), TUBORF‐K10R (**TUBORF‐K10R**), TUBORF‐K16R (**TUBORF‐K16R**) and TUBORF‐K(10/16)R (**TUBORF‐K(10/16)R**) or control (**pHAGE**). F) Western blotting analysis of SLC7A11 and GPX4 protein levels in lncTUBA3FP KO (**lncTUBA3FP KO #1**) or its control wild type (**WT**) cervical cancer cells transduced with lentivirus TUBORF‐WT, TUBORF‐K(10/16)R or its control pHAGE, and treated with Erastin (5 µm, 24 h). G) Western blotting analysis of SLC7A11 and GPX4 protein levels in lncTUBA3FP KO (**lncTUBA3FP KO #1**) or its control wild type (**WT**) cervical cancer cells transduced with lentivirus TUBORF‐**WT**, TUBORF‐K(10/16)Q or its control pHAGE, and treated with Erastin (5 µm, 24 h). H) Cells treated as in (F and G) were used to measure Fe^2+^ levels (*n* = 3). I) Cells treated as in (F and G) were used to examine GSH/GSSG levels (*n* = 3). J) Cells treated as in (F and G) were employed to measure lipid ROS levels (*n* = 3). K) Soft agar colony formation assay of cells treated as in (F and G). The representative images were photographed at 2 weeks after cell seeding. Magnification, ×100. Scar bars, 40 µm. L) Results were quantified in (K) (*n* = 6). Data were presented with mean ± SD. ^*^ *p* < 0.05, ^**^ *p* < 0.01, and ^***^ *p* < 0.001, Student's *t*‐test.

Post‐translational modifications (PTMs) regulate the activity, stability, and folding of proteins, as well as metabolism, signaling pathways, tumorigenesis, and other cellular biological processes.^[^ ^30^ ^]^ To investigate PTMs on TUBORF peptide, co‐immunoprecipitation assay (Co‐IP) was performed to analyze lysine acetylation (Kac), lactylation (Kla), succinylation (Ksucc), propionylation (Kpr), malonylation (Kmal), butyrylation (Kbu), hydroxyisobutyrylation (Khib), and crotonylation (Kcr) with a various of pan‐acyl‐lysine antibodies. We found that TUBORF was only modified by lysine acetylation (Kac) but not other acyl‐lysine modifications in CC cells (**Figure** **4**A). The endogenous IP further confirmed that TUBORF was acetylated in CC cells (Figure 4B,C). Since TUBORF has only two lysine sites, we generated a series of TUBORF mutants by replacing Lys (K) with Arg (R) or Gln (Q) (Figure 4D). Replacing Lysine (K) with Glutamine (Q) mimics acetylation, whereas replacing it with Arginine (R) simulates the non‐acetylated state. IP showed that mutation of K10, K16, or K(10/16) all inhibited the acetylation of TUBORF (Figure 4E), indicating that both K10 and K16 were the major acetylation sites of TUBORF. Next, we overexpressed WT, K(10/16)R mutant, or K(10/16)Q mutant TUBORF in lncTUBA3FP KO cells (Figure S4, Supporting Information). We found that mutation of K(10/16)R reduced the levels of SLC7A1, GPX4, GSH, and the sizes of soft agar colonies, and increased the levels of intracellular Fe^2+^ and lipid ROS when compared to the WT TUBORF group (Figure 4F,H–L). In contrast, mutation of K(10/16)Q further enhanced the effect of TUBORF on ferroptosis and tumorigenesis of CC cells (Figure 4H–L). These data suggest that acetylation of TUBORF inhibits ferroptosis and promotes the malignant proliferation of CC cells.

**Figure 5:**
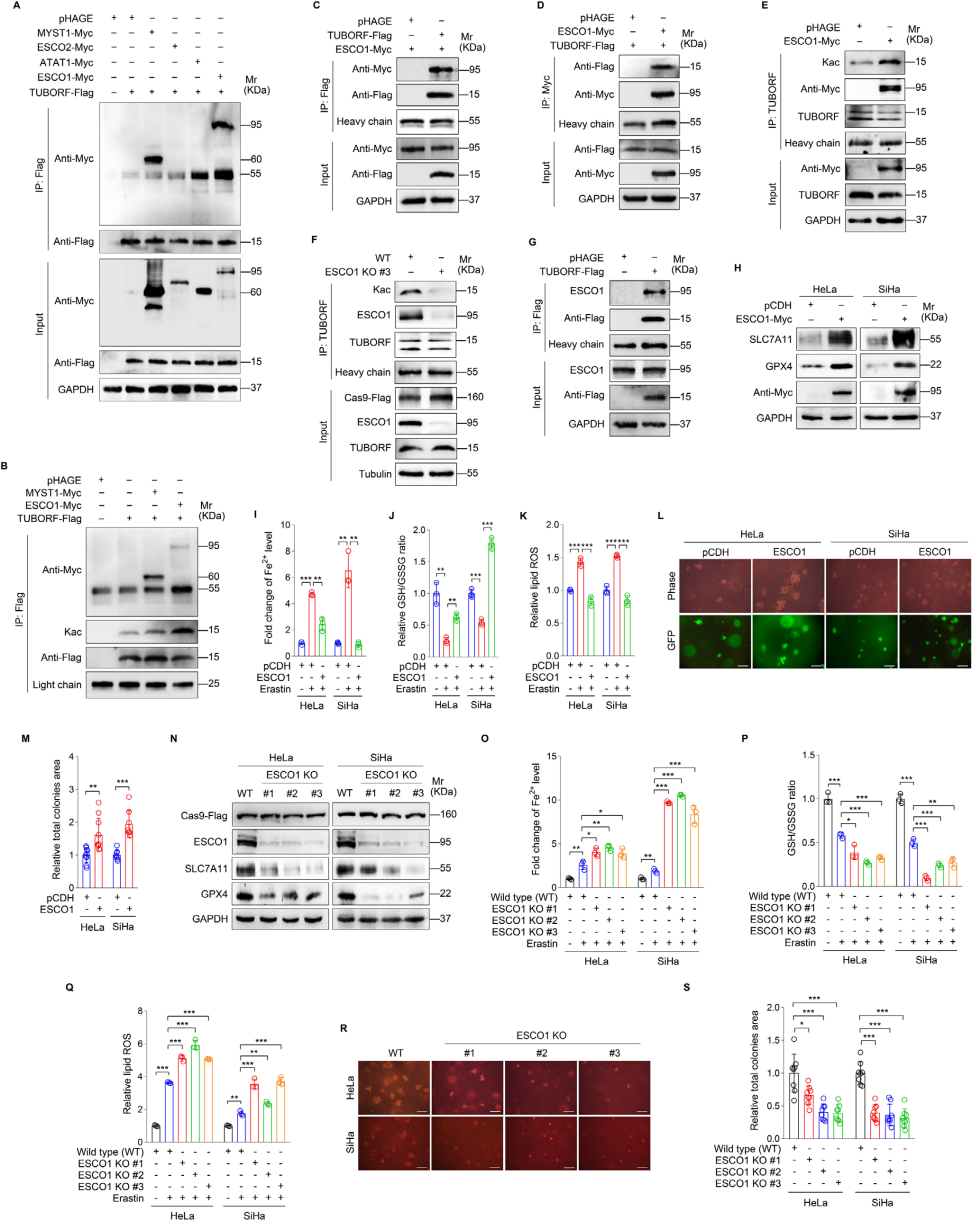
Acetyltransferase ESCO1 enhances TUBORF acetylation to inhibit ferroptosis and promote the malignant proliferation of cervical cancer cells. A) HeLa cells were co‐transduced by TUBORF‐Flag (**TUBORF‐Flag**) and MYST1‐Myc (**MYST1‐Myc**), ESCO2‐Myc (**ESCO2‐Myc**), ATAT1‐Myc (**ATAT1‐Myc**), ESCO1‐Myc (**ESCO1‐Myc)** or the control (**pHAGE**), respectively. Immunoprecipitation assay was performed with the anti‐Flag antibody to screen the acyltransferases interacted with TUBORF. B) IP assay of TUBORF acetylation in HeLa cells co‐transduced by TUBORF‐Flag (**TUBORF‐Flag**) and MYST1‐Myc (**MYST1‐Myc**) or ESCO1‐Myc (**ESCO1‐Myc**), respectively. C) ESCO1 (**ESCO1‐Myc**)‐overexpressing HeLa cells were infected with lentiviral TUBORF‐Flag (**TUBORF‐Flag**) or its control (**pHAGE**). An immunoprecipitation assay was performed to examine the interaction between TUBORF and ESCO1 with the anti‐Flag antibody. D) TUBORF‐Flag (**TUBORF‐Flag**)‐overexpressing HeLa cells were infected with lentiviral ESCO1‐Myc (**ESCO1‐Myc**) or its control (**pHAGE**). An immunoprecipitation assay was performed to detect the interaction between TUBORF and ESCO1 with the anti‐Myc antibody. E) IP assay of TUBORF acetylation in HeLa cells infected with lentiviral ESCO1 (**ESCO1‐Myc**) or its control (**pHAGE**) with the anti‐TUBORF antibody. F) IP assay of TUBORF acetylation in ESCO1 knockout cells (**ESCO1 KO #3**) with the anti‐TUBORF antibody in HeLa cells. G) IP assay of the interaction between ESCO1 and TUBORF in HeLa cells transduced with lentiviral TUBORF‐Flag (**TUBORF‐ORF**) or control (**pHAGE**) with the anti‐Flag antibody. H) Western blotting analysis of SLC7A11 and GPX4 expression levels in ESCO1‐Myc‐expression cervical cancer cells or its control (**pCDH**) after Erastin (5 µm, 24 h) treatment. I) Cells treated as in (H) were used to measure Fe^2+^ levels (*n* = 3). J) Cells treated as in (H) were employed to measure GSH/GSSG levels (*n* = 3). K) Cells treated as in (H) were used to examine lipid ROS levels (*n* = 3). L) Soft agar colony formation assay of cells treated as in (H). The representative images were photographed at two weeks after cell seeding. Magnification, ×100. Scar bars, 40 µm. M) Results were quantified in (L) (*n* = 8). N) Western blot analysis of SLC7A11 and GPX4 expression levels in ESCO1 knockout (**ESCO1 KO #1‐3**) or its control cells (**WT**) after Erastin (5 µm, 24 h) treatment. O) Cells treated as in (N) were used to measure Fe^2+^ levels (*n* = 3). P) Cells treated as in (N) were employed to measure GSH/GSSG levels (*n* = 3). Q) Cells treated as in (N) were used to examine lipid ROS levels (*n* = 3). R) Soft agar colony formation assay of cells treated as in (N). The representative images were photographed two weeks after cell seeding. Magnification, ×100. Scar bars, 40 µm. S) Results were quantified in (R) (*n* = 8). Data were presented with mean ± SD. ^*^ *p* < 0.05, ^**^ *p* < 0.01, and ^***^ *p* < 0.001, Student's *t*‐test.

Protein lysine acetylation is usually regulated by acetyltransferase and/or deacetyltransferase.^[^ ^31^ ^]^ To determine the critical acetyltransferase that primarily acetylates the lysine residues in TUBORF, Co‐IP was performed in CC cells. We found that TUBORF interacted with MYST1 and ESCO1 (**Figure** **5**A; Figure S5, Supporting Information). However, only ESCO1 overexpression increased the acetylation level of TUBORF (Figure 5B). The exogenous Co‐IP further confirmed the interaction between TUBORF and ESCO1 (Figure 5C,D). Importantly, ESCO1 overexpression effectively increased the acetylation level of endogenous TUBORF, while ESCO1 knockdown exhibited an opposite effect (Figure 5E,F; Figure S6, Supporting Information). We immunoprecipitated Flag‐TUBORF in TUBORF‐overexpressed cells, and found that endogenous ESCO1 protein was also detected (Figure 5G). Then, we examined the effect of ESCO1 on ferroptosis and the malignant proliferation of CC cells. ESCO1 overexpression dramatically increased the levels of SLC7A11, GPX4, GSH, and the sizes of soft agar colonies, and decreased the levels of intracellular Fe^2+^ and lipid ROS of CC cells (Figure 5H–M). Consistently, ESCO1 knockdown exhibited the opposite effects (Figure 5N–S).

To confirm whether the function of ESCO1 on ferroptosis is primarily mediated through acetylated TUBORF, we overexpressed ESCO1 in TUBORF‐WT or K(10/16)R mutant cells. We found that ESCO1 overexpression led to an increase in the levels of SLC7A11, GPX4, and GSH, as well as decreased levels of intracellular Fe^2+^ and lipid ROS in TUBORF‐WT cells, but not in TUBORF‐K(10/16)R cells (Figure S7, Supporting Information), indicating that the function of ESCO1 on ferroptosis was achieved by acetylated TUBORF.

**Figure 6:**
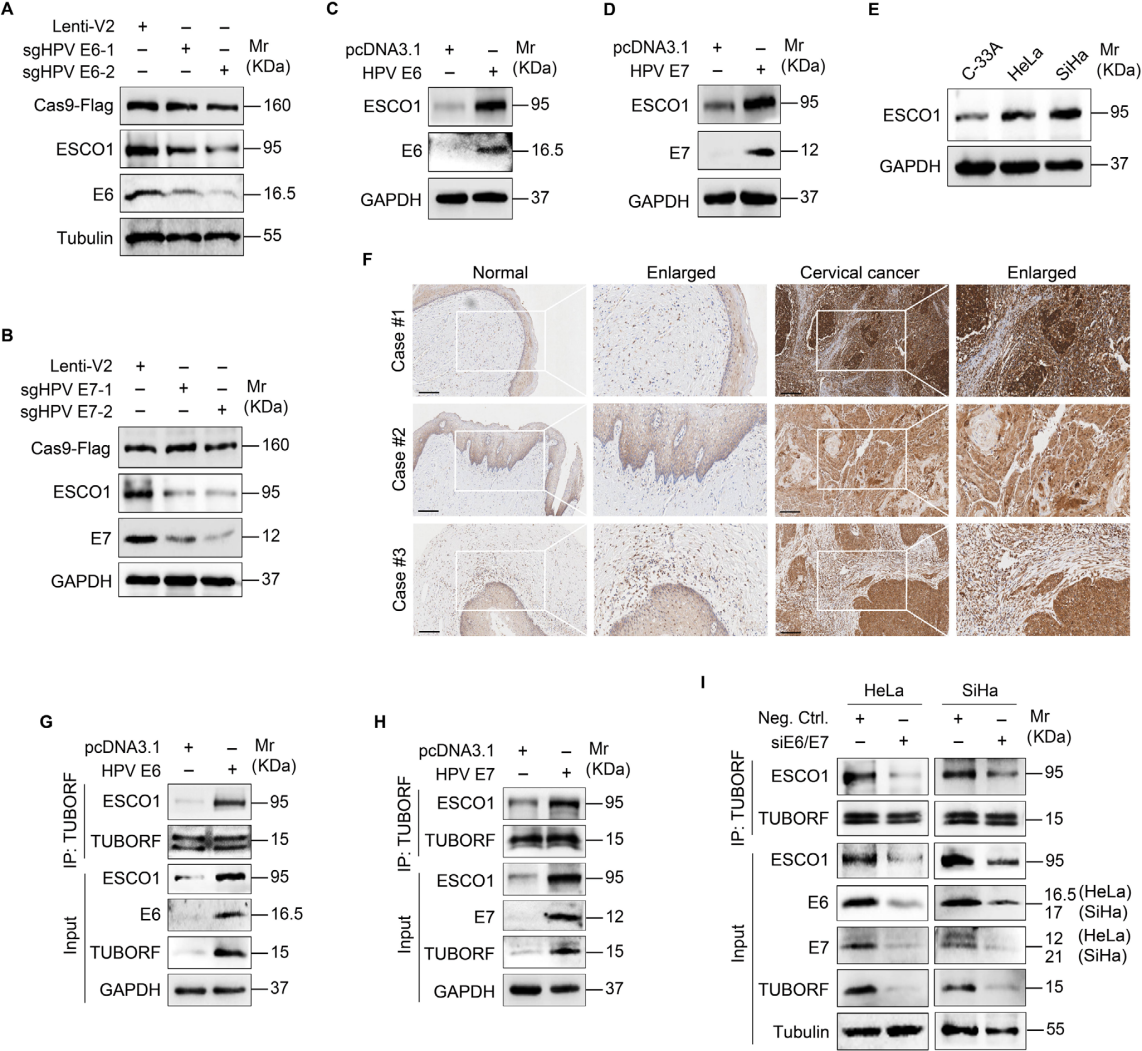
ESCO1 is upregulated by HPV E6 and E7. A) Western blotting analysis of the expression of ESCO1 in HeLa cells infected with lentiviral sgHPV E6‐1 (**sgHPV E6‐1**), sgHPV E6‐2 (**sgHPV E6‐2**) or control (**Lenti‐V2**). B) Western blotting analysis of the expression of ESCO1 in HeLa cells infected with lentiviral sgHPV E7‐1 (**sgHPV E7‐1**), sgHPV E7‐2 (**sgHPV E7‐2**) or control (**Lenti‐V2**). C) HPV‐negative cervical cancer cells C‐33A were transfected with HPV E6 (**HPV E6**) construct or its control (**pcDNA3.1**). Western blotting was performed to examine the ESCO1 expression level. D) HPV‐negative cervical cancer cells C‐33A were transfected with HPV E7 (**HPV E7**) construct or its control (**pcDNA3.1**). Western blotting was performed to examine the ESCO1 expression level. E) ESCO1 protein levels were detected in HPV‐negative cervical cancer cells (**C‐33A**) and HPV‐positive cervical cancer cells (**HeLa, SiHa**). F) Representative images of immunohistochemistry staining (IHC) for ESCO1 expression in cervical cancer tissues and their corresponding normal cervical epithelial tissues. Scar bars, 100 µm. G) IP assay of the interaction between TUBORF and ESCO1 in C‐33A cells transfected with HPV E6 construct or its control pcDNA3.1 with the anti‐TUBORF antibody. H) IP assay of the interaction between TUBORF and ESCO1 in C‐33A cells transfected with HPV E7 construct or its control pcDNA3.1 with the anti‐TUBORF antibody. I) IP assay of the interaction between TUBORF and ESCO1 in HeLa cells transfected with an HPV E6/E7 siRNA pool (**siE6/E7**) with the anti‐TUBORF antibody.

To determine whether HPV oncoproteins E6 and E7 regulate the expression of ESCO1, we knocked down E6 and E7 in HPV‐positive CC cells. Western blotting showed that the silence of E6 and E7 decreased ESCO1 levels (**Figure** **6**A,B). Conversely, overexpression of E6 and E7 increased ESCO1 levels in HPV‐negative C‐33A cells (Figure 6C,D). ESCO1 protein level was upregulated in HPV‐positive CC cells HeLa and SiHa, compared to that in HPV‐negative cells C‐33A (Figure 6E). IHC staining confirmed that ESCO1 expression was higher in CC tissues than in normal cervical epithelial tissues (Figure 6F).

Next, we explored whether ESCO1 promotes the acetylation of TUBORF mediated by E6 or E7. Co‐IP showed that overexpression of E6 and E7 strengthened the interaction between the endogenous ESCO1 and TUBORF (Figure 6G,H). Conversely, the knockdown of E6 and E7 weakened the interaction between ESCO1 and TUBORF (Figure 6I). To determine whether E6/E7‐upregulated lncTUBA3FP affects the abundance of ESCO1 or the interaction between ESCO1 and TUBORF, we performed an immunoprecipitation assay in HeLa cells transduced with lncTUBA3FP or lncTUBA3FP_Mut. We showed that overexpression of lncTUBA3FP or lncTUBA3FP_Mut did not alter ESCO1 expression level, compared to their control pCDH (Figure S8A, Supporting Information). Also, overexpression of lncTUBA3FP_Mut had no effect on the interaction between ESCO1 and TUBORF (Figure S8B, Supporting Information), indicating that lncTUBA3FP does not influence the protein abundance of ESCO1 nor its binding affinity for TUBORF.

Taken together, these results suggest that HPV oncoproteins E6 and E7 upregulate and recruit ESCO1 to bind to and acetylate TUBORF, leading to inhibition of ferroptosis and promotion of the malignant proliferation of CC cells.

### 2.5 Acetylated TUBORF Promotes IRGQ Protein Degradation that Inhibits Ferroptosis to Enhance the Malignant Proliferation of Cervical Cancer Cells

**Figure 7:**
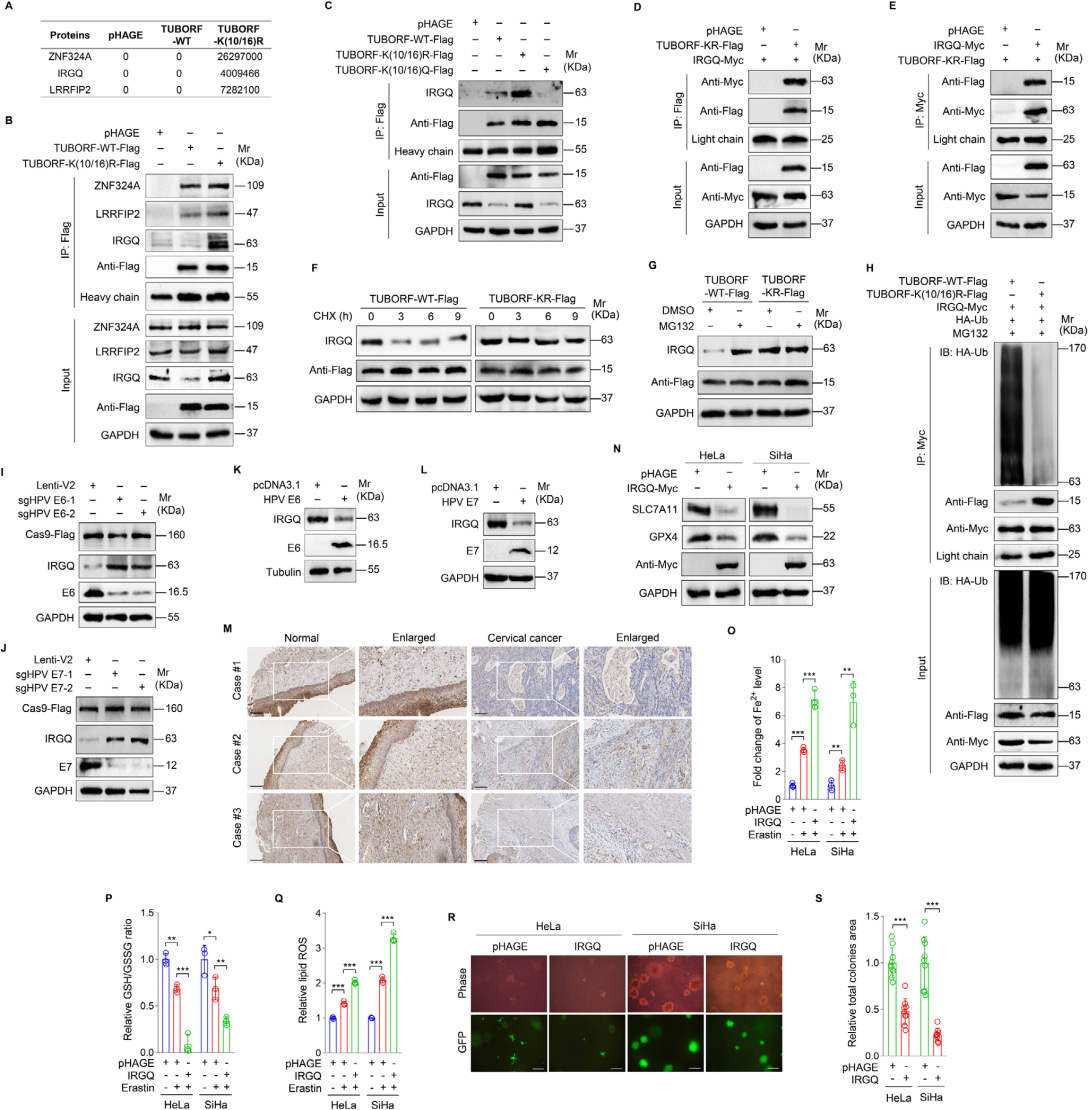
Acetylated TUBORF promotes IRGQ protein degradation to suppress ferroptosis and enhance the malignant proliferation of cervical cancer cells. A) Mass spectrometry analysis of the differential binding proteins in TUBORF‐WT‐ and TUBORF‐K(10/16)R‐overexpressing HeLa cells. B) IP assay of the interaction between TUBORF‐K(10/16)R and ZNF324A, LRRFIP2, or IRGQ with the anti‐Flag antibody in HeLa cells. C) IP assay of the interaction between IRGQ and TUBORF‐K(10/16)R or TUBORF‐K(10/16)Q with anti‐Flag antibody in HeLa cells. D) IRGQ‐Myc‐overexpressing cells were transduced with lentiviral TUBORF‐K(10/16)R‐Flag (**TUBORF‐KR‐Flag**) or pHAGE. An immunoprecipitation assay was performed to examine the interaction between IRGQ and TUBORF‐KR with the anti‐Flag antibody in HeLa cells. E) TUBORF‐K(10/16)R‐Flag‐expression cells (**TUBORF‐KR‐Flag**) were co‐transduced with IRGQ‐Myc or its control pHAGE. An immunoprecipitation assay was performed to examine the interaction between IRGQ and TUBORF‐KR with the anti‐Myc antibody in HeLa cells. F) HeLa cells transduced with wild‐type TUBORF (**TUBORF‐WT‐Flag**) or K10/16R mutant TUBORF (**TUBORF‐KR‐Flag**) were treated with CHX (20 µg mL^−1^) for 0, 3, 6 and 9 h, respectively. Western blotting was performed to examine IRGQ expression. G) HeLa cells transduced with wild‐type TUBORF (**TUBORF‐WT‐Flag**) or K10/16R mutant TUBORF (**TUBORF‐KR‐Flag**) were treated with MG132 (10 µm) for 8 h. Western blotting was performed to detect IRGQ expression. H) HeLa cells treated as in (G) were co‐transfected with the HA‐Ub and IRGQ‐Myc constructs. An immunoprecipitation assay was performed to examine the level of IRGQ ubiquitination with the anti‐Myc antibody. I) Western blotting analysis of the IRGQ protein level in lentiviral sgHPV E6‐1 (**sgHPV E6‐1**), sgHPV E6‐2 (**sgHPV E6‐2**), or its control (**Lenti‐V2**) transduced HeLa cells. J) Western blotting analysis of the IRGQ protein level in lentiviral sgHPV E7‐1 (**sgHPV E7‐1**), sgHPV E7‐2 (**sgHPV E7‐2**), or its control (**Lenti‐V2**) transduced HeLa cells. K) Western blotting was performed to examine the IRGQ protein level in C‐33A cells transfected with HPV E6 construct (**HPV E6**) or its control (**pcDNA3.1**). L) Western blotting was performed to examine the IRGQ protein level in C‐33A cells transfected with HPV E7 construct (**HPV E7**) or its control (**pcDNA3.1**). M) Representative IHC images of ESCO1 expression in cervical cancer tissues and their paired normal cervical epithelial tissues. Scar bars, 100 µm. N) Western blotting analysis of SLC7A11 and GPX4 protein levels in Erastin‐treated HeLa and SiHa cells transduced with lentiviral IRGQ‐Myc (**IRGQ‐Myc**) or control (**pHAGE**). O) Cells treated as in (N) were used to measure Fe^2+^ levels (*n* = 3). P) Cells treated as in (N) were employed to measure GSH/GSSG levels (*n* = 3). Q) Cells treated as in (N) were used to examine lipid ROS levels (*n* = 3). R) Soft agar colony formation assay of cells treated as in (N). The representative images were photographed at 2 weeks after cell seeding. Magnification, ×100. Scar bars, 40 µm. S) Results were quantified in (R) (*n* = 9). Data were presented with mean ± SD. ^*^ *p* < 0.05, ^**^ *p* < 0.01, and ^***^ *p* < 0.001, Student's *t*‐test.

To elucidate the mechanism underlying TUBORF acetylation‐mediated ferroptosis and tumorigenesis, we treated cells with cycloheximide (CHX), a *de novo* protein biosynthesis inhibitor, and found that TUBORF acetylation did not affect its stability (Figure S9A, Supporting Information). Meanwhile, TUBORF acetylation had no effect on its subcellular localization (Figure S9B, Supporting Information). To determine whether TUBORF acetylation impacts protein interaction, mass spectrometry was performed to examine the differential binding proteins between TUBORF‐WT and TUBORF‐K(10/16)R‐overexpressing cells. The top three TUBORF‐K(10/16)R‐binding proteins were selected (**Figure** **7**A), and Co‐IP was further performed to reveal that IRGQ protein bound to TUBORF‐K(10/16)R, but not TUBORF‐WT (Figure 7B) or TUBORF‐K(10/16)Q mutant (Figure 7C). The exogenous Co‐IP also showed that IRGQ interacted with deacetylated TUBORF (Figure 7D,E).

Overexpression of TUBORF‐WT reduced IRGQ level compared to that of TUBORF‐K(10/16)R (Figure 7B,C), but no difference in IRGQ mRNA levels (Figure S10, Supporting Information). To determine whether deacetylated TUBORF influences the stability of IRGQ protein, we treated TUBORF‐WT and TUBORF‐K(10/16)R transduced cells with CHX. We found that TUBORF‐K(10/16)R inhibited TUBORF‐WT‐mediated IRGQ protein degradation (Figure 7F; Figure S11A, Supporting Information). TUBORF‐WT‐mediated IRGQ protein degradation was completely suppressed by the protease inhibitor MG132 (Figure 7G; Figure S11B, Supporting Information). As expected, TUBORF‐K(10/16)R inhibited the ubiquitination degradation of IRGQ protein (Figure 7H). These results suggest that TUBORF acetylation promotes IRGQ protein degradation via a ubiquitin‐proteasome pathway.

To examine whether HPV oncoproteins E6 and E7 influence the expression of IRGQ, we knocked down E6 and E7 and found an obvious increase in IRGQ protein expression (Figure 7I,J). Conversely, E6 and E7 overexpression achieved an opposite effect in C‐33A cells (Figure 7K,L). IRGQ protein was downregulated in CC tissues (Figure 7M). To determine the role of IRGQ in ferroptosis and tumorigenesis, we overexpressed IRGQ in CC cells and observed a significant inhibition of SLC7A11 and GPX4 expression, a decrease of GSH content, an increase of intracellular Fe^2+^ content and lipid ROS level, as well as a shrinkage of soft agar colonies (Figure 7N–S). To confirm whether the effect of TUBORF on ferroptosis is predominantly achieved by IRGQ ubiquitination, we performed mass spectrometry analysis and identified K379 as a potential ubiquitination modification site on IRGQ (Figure S12A, Supporting Information). Subsequently, the mutation of K379 was sufficient to abolish the ubiquitination degradation of IRGQ induced by TUBORF‐WT (Figure S12B,C, Supporting Information). Furthermore, overexpression of TUBORF in IRGQ‐WT cells decreased the levels of intracellular Fe^2+^ and lipid ROS, and increased the levels of GSH but not in IRGQ_379KR cells (Figure S12D–F, Supporting Information). These data demonstrated that the function of TUBORF on ferroptosis was achieved by modulating IRGQ ubiquitination.

### 2.6 Silencing ESCO1 or TUBORF Amplifies Anticancer Effect by Paclitaxel Both in Cervical Cancer Cells and in vivo

**Figure 8:**
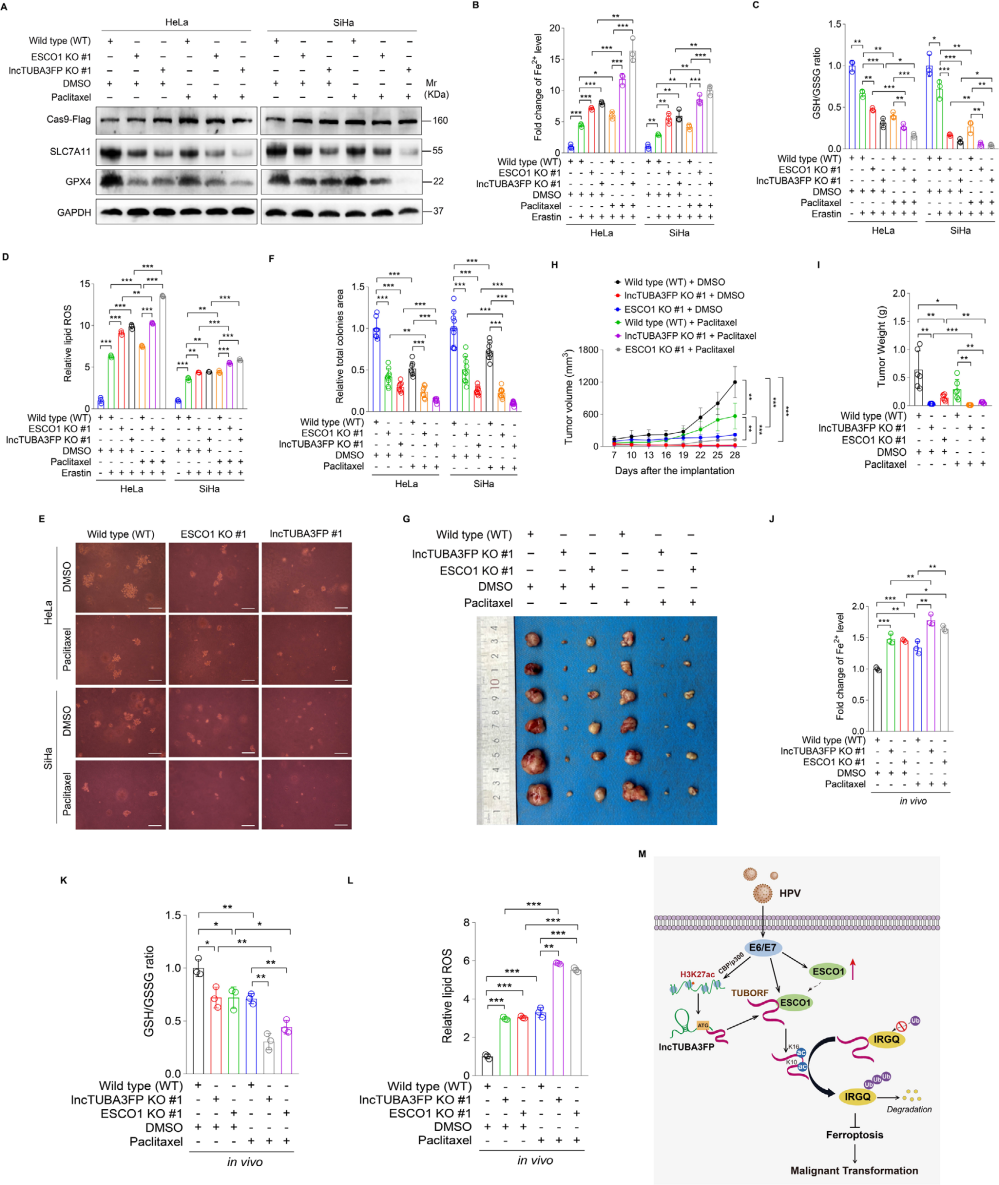
Knockdown of ESCO1 or TUBORF enhances paclitaxel‐initiated cell ferroptosis and amplifies the anticancer effect of paclitaxel in cervical cancer. A) ESCO1 knockout cells (**ESCO1 KO #1**), lncTUBA3FP knockout cells (**lncTUBA3FP KO #1**), or the control (**WT**) were treated with 5 µm Erastin, 10 µm paclitaxel or DMSO for 24 h. Cells were collected for Western blotting analysis of SLC7A11 and GPX4 protein levels. B) Cells treated as in (A) were employed to measure Fe^2+^ levels (*n* = 3). C) Cells treated as in (A) were used to examine GSH/GSSG levels (*n* = 3). D) Cells treated as in (A) were used to measure lipid ROS levels (*n* = 3). E) Soft agar colony formation assay of cells treated as in (A). The representative images were photographed two weeks after cell seeding. Magnification, ×100. Scar bars, 40 µm. F) Results were quantified in (E) (*n* = 11). G) Representative isolated tumor images from nude mice with paclitaxel or DMSO treatment and injected with ESCO1 knockout (**ESCO1 KO #1**), lncTUBA3FP knockout (**lncTUBA3FP KO #1**), and wild type (**WT**) transduced SiHa cells after 28 days were shown (*n* = 6). H) Tumor volumes in (G) were measured every 3 days starting from day 7 (*n* = 6). I) Tumor weights in (G) were recorded after the isolation of tumors (*n* = 6). J) Tissues treated as in (G) were employed to measure Fe^2+^ levels, with a portion of each tissue being selected for combined detection (*n* = 3). K) Tissues treated as in (G) were used to examine GSH/GSSG levels, with a portion of each tissue being selected for combined detection (*n* = 3). L) Tissues treated as in (G) were used to measure lipid ROS levels, with a portion of each tissue being selected for combined detection (*n* = 3). M) A schematic working model of HPV‐induced TUBORF acetylation. HPV oncoproteins E6 and E7 upregulate lncTUBA3FP level by histone H3K27ac modification subjected to acetyltransferase CBP/p300, which encodes a peptide named TUBORF. E6 and E7 increase and recruit acetyltransferase ESCO1 to bind to and acetylate TUBORF. Acetylated TUBORF binds to IRGQ protein and promotes its degradation, resulting in inhibition of ferroptosis and enhancement of the malignant proliferation of cervical cancer. Data were presented with mean ± SD. ^*^ *p* < 0.05, ^**^ *p* < 0.01, and ^***^ *p* < 0.001, Student's *t*‐test.

Paclitaxel, a highly effective chemotherapeutic agent utilized in the treatment of CC, has been recognized for its repression role in tumor progression in various cancer types through ferroptosis.^[^ ^32^ ^]^ Considering the elevated expression of ESCO1 and TURORF and their involvement in the regulation of ferroptosis in CC cells, we sought to investigate whether silencing ESCO1 or TURORF influences the anti‐tumor effect of paclitaxel. CC cells with ESCO1 or lncTUBA3FP knockout were treated with paclitaxel or DMSO as control. Silence of ESCO1 or lncTUBA3FP not only strengthened paclitaxel‐induced reduction levels of GPX4 and SLC7A11 proteins, and GSH content but also promoted increased intracellular Fe^2+^ content and lipid ROS levels induced by paclitaxel compared with paclitaxel‐untreated cells (**Figure** **8**A–D). More importantly, the knockout of ESCO1 or lncTUBA3FP enhanced the ability of paclitaxel to inhibit the malignant proliferation of CC cells (Figure 8E,F). These findings indicate that targeting ESCO1 or TUBORF peptide in CC cells enhances the anticancer effect of paclitaxel in vitro.

Meanwhile, we silenced ESCO1 in cells overexpressing lncTUBA3FP and observed that the knockdown of ESCO1 resulted in a substantial increase in intracellular Fe^2+^ levels and lipid ROS production, accompanied by a decrease in GSH content (Figure S13A–C, Supporting Information). Consistently, following ESCO1 knockdown, the size of soft agar colonies formed by these lncTUBA3FP‐overexpressing cells decreased (Figure S13D, Supporting Information). These data suggest that the knockdown of ESCO1 rescues lncTUBA3FP‐mediated ferroptosis inhibition and promotion of malignant proliferation, both in the presence and absence of paclitaxel treatment.

Next, we sought to determine whether TUBORF and ESCO1 regulate the anticancer activity of paclitaxel in vivo. TUBORF‐ or ESCO1‐silenced SiHa cells were implanted subcutaneously into the right flanks of nude mice. After 7 days, mice were divided randomly into two groups and subsequently treated with DMSO or paclitaxel. As expected, silencing TUBORF or ESCO1 potentiated the ability of paclitaxel to inhibit tumor weight and size (Figure 8G–I). In addition, silencing of ESCO1 or lncTUBA3FP not only exacerbated the paclitaxel‐mediated decrease in GSH content but also augmented the paclitaxel‐induced increase in Fe^2+^ and lipid ROS levels, when compared to mice solely treated with paclitaxel (Figure 8J–L). These results collectively suggest that targeting TUBORF or ESCO1 strengthens the anticancer activity of paclitaxel by inducing ferroptosis in cervical cancer in vivo.

## 3 Discussion

Although lncRNAs have ever been recognized as non‐coding RNAs,^[^ ^33^ ^]^ new analytical methods and techniques uncover that some lncRNAs contain ORFs, which can encode functional peptides.^[^ ^34^ ^]^ Studies have shown that lncRNA‐encoded peptides are implicated in immune inflammation, cancer development, and cell signaling.^[^ ^1^, ^10^, ^35^ ^]^ However, whether lncRNA‐encoded peptides exist to regulate the malignancy of cervical cancer is still unknown. In this study, we revealed that TUBORF, a never‐reported peptide, is encoded by a previously annotated lncRNA, lncTUBA3FP. Both TUBORF and lncTUBA3FP were highly expressed in cervical cancer tissues. Particularly, TUBORF, rather than linear lncTUBA3FP or the parental gene of lncTUBA3FP, *LRRC74B*, inhibited ferroptosis and promoted the malignant proliferation of cervical cancer cells. Consequently, these novel findings shed unprecedented light on the role of lncRNA‐encoded peptides in the malignancy of cervical cancer, which refreshes the current understanding of the function of lncRNA‐encoded peptides in tumorigenesis. As for lncRNA and cervical cancer, we have previously demonstrated that HPV E6 and E7 downregulated lncRNA‐CCDST to enhance cervical cancer oncogenicity by promoting its binding protein DHX9 degradation.^[^ ^26^ ^]^ Here, we further showed that HPV E6 and E7 upregulated lncTUBA3FP through CBP/p300‐mediated H3K27ac of lncTUBA3FP enhancer, resulting in the high expression of TUBORF encoded by lncTUBA3FP in cervical cancer. Since other enzymes responsible for H3K27ac, such as HDAC1, have also been reported,^[^ ^30b^ ^]^ the possibility that these enzymes may be involved in H3K27ac of lncTUBA3FP enhancer cannot be excluded, therefore, further research is needed.

Over the past decade, more than 400 types of PTMs have been identified. PTMs are the engine of many cellular signaling events, including autophagy, ferroptosis, metabolism, and tumorigenesis.^[^ ^26^, ^36^ ^]^ As a representative and routine PTMs, lysine acetylation modification influences various physiological processes and signaling pathways in a dynamic manner.^[^ ^30^, ^37^ ^]^ In the current study, we showed that lncTUBA3FP‐encoded TUBORF peptide could be acetylated both endogenously and exogenously. Subsequently, acetylated TUBORF functioned as an oncogenic activator to promote the malignant proliferation of cervical cancer cells by suppressing ferroptosis. Lysine acetylation modification is usually regulated by acetyltransferase and/or deacetyltransferase.^[^ ^38^ ^]^ We illustrated that ESCO1 could interact with and acetylate TUBORF. As a lysine acetyltransferase, ESCO1 is highly expressed in a variety of tumors, including bladder cancer, prostate cancer, and endometrial cancer;^[^ ^36^, ^39^ ^]^ however, its role in cervical cancer has not been elucidated. Currently, we indicated that ESCO1 was highly expressed in cervical cancer tissues. Upregulation of ESCO1 inhibited ferroptosis and promoted the malignant proliferation of cancer cells. Conversely, the knockout of ESCO1 exhibited an opposite effect. In terms of upstream mechanisms, although we showed that HPV E6 and E7 positively regulated ESCO1 expression, the underlying mechanism remains unclear. Since protein acetylation and deacetylation are a balanced process in vivo, whether there is a deacetyltransferase responsible for TUBORF acetylation also requires further investigation.

It is widely recognized that E6 and E7 are the primary transforming oncoproteins of HPV and are essential for the development and maintenance of malignant tumors. Numerous studies have highlighted that HPV E6 and E7 collaboratively regulate key cellular functions, including the cell cycle and apoptosis, ultimately resulting in cellular immortalization and malignant proliferation.^[^ ^40^ ^]^ Here, we further implied a synergistic function of E6 and E7 to upregulate the expression level of lncTUBA3FP through histone H3K27ac modification facilitated by CBP/p300. Furthermore, E6 and E7 enhanced and recruited acetyltransferase ESCO1 to bind to and acetylate TUBORF. The underlying mechanism is that E6 and E7 may indirectly modulate the expression of ESCO1 and TUBORF through independent strategies, ultimately converging on the shared objective of promoting tumorigenesis.

A functional gene belonging to the GTP enzyme family, IRGQ, plays a role in various inflammatory and autoimmune diseases.^[^ ^24^, ^41^ ^]^ In the current study, we found that IRGQ was downregulated in cervical cancer tissues, and interacted with deacetylated TUBORF to prevent its degradation. HPV E6‐ and E7‐induced TUBORF acetylation reduced the stability of IRGQ protein. Subsequently, the downregulation of IRGQ inhibited ferroptosis to promote the malignant proliferation of cervical cancer cells. Thus, our findings provide an innovative insight into the role of IRGQ in the malignancy of cervical cancer. Although acetylated TUBORF was shown to bind to IRGQ protein, resulting in its degradation via a ubiquitin‐proteasome pathway, the presence of ubiquitination ligase or deubiquitination enzyme mediated its degradation process remains to be further determined. Moreover, our findings revealed that overexpression of IRGQ modulated the expression of SLC7A11 and GPX4 proteins, ultimately enhancing ferroptosis. Nevertheless, the underlying mechanism remains unknown, while transcription inhibition or acceleration of the degradation process for SLC7A11 and GPX4 proteins may be involved. Additionally, IRGQ may directly or indirectly impact the other critical proteins involved in iron uptake and export, consequently causing variations in the levels of ferroptosis.

Taxane‐based drugs, such as paclitaxel, remain the first‐line chemotherapeutic treatment for cervical cancer. However, the emergence of chemoresistance, peripheral neuropathy, nephrotoxicity, and hepatorenal toxicity to paclitaxel has posed significant challenges in cervical cancer treatment.^[^ ^42^ ^]^ Here we showed that silence of either lncTUBA3FP or ESCO1 enhanced the sensitivity of cervical cancer cells to paclitaxel, as evidenced by reduced tumorigenesis and increased ferroptosis levels. These findings suggest that targeting TUBORF peptide or ESCO1 may represent a promising therapeutic strategy for the treatment of cervical cancer.

In summary, TUBORF, a peptide encoded by lncTUBA3FP, inhibits ferroptosis and increases the malignant proliferation of cervical cancer cells. HPV E6 and E7 promote lncTUBA3FP expression through histone acetyltransferase CBP/p300‐mediated H3K27ac of lncTUBA3FP enhancer. E6 and E7 also elevate acetyltransferase ESCO1 expression and facilitate the interaction between ESCO1 and TUBORF to increase the acetylation of TUBORF. Acetylated TUBORF enhances the degradation of IRGQ protein via a ubiquitin‐proteasome pathway (Figure 8M). The novel discovery of TUBORF opens an avenue for the research of cervical cancer based on lncRNA‐encoded peptide and its modification that may act as potential therapeutic targets.

## 4 Experimental Section

### Ethics Statement

The clinical studies in this research were reviewed and ethically approved by the Institutional Ethics Committee of Nanjing Women and Children's Healthcare Hospital Nanjing, Nanjing Medical University, and by the Institutional Ethics Committee of the First Affiliated Hospital of Nanjing Medical University.

### Clinical Samples

Matched cervical cancer tissues and normal tissues were collected from Nanjing Women and Children's Healthcare Hospital, Nanjing Medical University. Tissue microarray chips containing 287 cases of cervical primary squamous cell carcinoma and 146 cases of normal squamous epithelium were collected from the First Affiliated Hospital of Nanjing Medical University. Written informed consent was acquired from all patients.

### Cell Culture and Treatment

C‐33A, HT‐3, HeLa, SiHa, HEK293T, and UPCI‐SCC‐154 cell lines were cultured in Dulbecco's modified Eagle's medium (DMEM) with 10% fetal bovine serum (FBS), 1% penicillin, 1% streptomycin and 1% gentamicin. CAL‐27 was cultured in Minimum Essential Medium (MEM) with 10% FBS, 1% penicillin, 1% streptomycin, and 1% gentamicin. UPCI‐SCC‐090 was cultured in MEM with 10% FBS, 1 mm Sodium Pyruvate, 1% penicillin, 1% streptomycin, and 1% gentamicin. SCC‐9 was cultured in DMEM/F12 Medium with 10% FBS, 400 ng mL^−1^ Hydrocortisone, 1% penicillin, 1% streptomycin, and 1% gentamicin. CAL‐27, SCC‐9, and UPCI‐SCC‐090 cell lines were purchased from Shanghai Zhongqiao Xinzhou Biotechnology (Shanghai, China). UPCI‐SCC‐154 was purchased from Zhejiang Meisen Cell Technology Company (Hangzhou, China). All cell lines were cultivated in an atmosphere of 5% CO_2_, and 99% relative humidity at 37 °C, and were examined for mycoplasma contamination using Myco‐Blue Mycoplasma Detector (Vazyme). The cells were respectively treated with 20 µg mL^−1^ Cycloheximide (CHX) (Cell Signal Technology) for different time‐points, proteasome inhibitor MG132 (Selleck) at 10 µm for 6 h, ferroptosis inducer Erastin (MCE) for 24 h, or CBP/p300 catalytic inhibitor A‐485 at 10 µm for 24 h. siRNA oligonucleotides targeting HPVs E6/E7 were purchased from GenePharma as previously described.^[^ ^26^ ^]^

### Lentiviral Packaging of Plasmid and Infection

TUBORF‐Flag/HA, TUBORF‐K10R, TUBORF‐K16R, TUBORF‐K(10/16)R, TUBORF‐K(10/16)Q, TUBORF‐5′UTR + ORF‐Flag, TUBORF‐5′UTR + ORF_Mut‐Flag and IRGQ‐Myc were cloned into pHAGE‐CMV‐MCSIzsGreen (pHAGE for short). The skeleton plasmid of lentiviral vectors lncRNA TUBA3FP, lncRNA TUBA3FP_Mut, MYST1‐Myc, ATAT1‐Myc, ESCO1‐Myc, ESCO2‐Myc, KAT2A‐Flag, KAT2B‐Flag, KAT5‐Flag and MYST2‐Flag was pCDH‐CMV‐MCS‐EF1‐copGFP (pCDH for short). HPV E6‐HA, HPV E7‐HA, CBP‐HA, and p300‐HA were loaded onto the pcDNA3.1 vector. The sgRNAs targeting HPV E6/E7, lncTUBA3FP, and ESCO1 were designed and inserted into lentiviral vector Lenti‐CRISPR‐V2. Sequences of sgRNAs are summarized in Table S2 (Supporting Information). All mutant plasmids were generated using a Mut Express II Fast Mutagenesis Kit V2 (Vazyme, China). Plasmids and sgRNAs were co‐transfected with psPAX2 and pMD2.G to generate lentiviruses as previously described.^[^ ^43^ ^]^ Plasmid transfection was performed using Lipofectamine 2000 (Life Technologies). Lentiviruses were collected to infect cervical cancer cell lines.

### Western Blotting and Antibodies

The whole‐cell lysates were extracted for Western blotting as previously described.^[^ ^36^, ^44^ ^]^ Protein lysates were separated using 15% Tris‐SDS‐polyacrylamide gel electrophoresis (PAGE) and then electroblotted onto a Nitrocellulose blotting membrane (GE Whatman, Britain England) of 0.22 µm. The target proteins were incubated in specific primary antibodies. Anti‐GAPDH, anti‐Tubulin, anti‐HPV E6, and anti‐HPV E7 were purchased from Santa Cruz Biotechnology (Dallas, TX, USA). Anti‐Flag, anti‐Myc, and anti‐HA were obtained from MEDICAL & BIOLOGICAL LABORATORIES CO., LTD. (Seoul, Korea); anti‐IRGQ, anti‐SLC7A11, and anti‐GPX4 were from Proteintech Group, Inc (Wuhan, China); anti‐ESCO1 was from HUABIO (Hangzhou, China). For TUBORF polyclonal antibody generation, the recombinant TUBORF (1‐92 aa) was expressed and purified using the prokaryotic expression system. Three experimental Japanese white rabbits were immunized. The serum was purified by antigen‐affinity purification.

### Quantitative Real‐Time PCR Analysis (RT‐qPCR) of mRNA Transcripts

Total RNA was extracted using TRIzol total RNA isolation reagent (Life Technologies, Grand Island, NY, USA). cDNAs were acquired by reverse transcription using HiScript Q RT SuperMix (Vazyme, Nanjing, China). The RT‐qPCR assay was performed using AceQ qPCR SYBR Green Master Mix (Vazyme, Nanjing, China). The sequences of the primers for GAPDH included 5′‐GAAGGTGAAGGTCGGAGTC‐3′ (forward) and 5′‐GAAGATGGTGATGGGATTTCC‐3′ (reverse). Primers specific for the lncTUBA3FP gene were 5′‐CCTGCTGCATGTTGTACAGA‐3′ (forward) and 5′‐TAAATCCAATCGGACACCAGT‐3′ (reverse). Primers specific for the IRGQ gene were 5′‐CGCCTGCTTCGGTCCCCACA‐3′ (forward) and 5′‐GCACGGTCCAGAGCACCACA‐3′ (reverse). Primers specific for the LRRC74B gene were 5′‐TGCCGCCCTCACAGTGAACCAG‐3′ (forward) and 5′‐TGGGCCGCCTGCTCCTCCAG‐3′ (reverse).

### Chromatin Immunoprecipitation (ChIP) Assay

EZ‐Magna ChIP A/G Chromatin Immunoprecipitation Assay Kit (Merck, Darmstadt, Germany) was used for ChIP analysis as previously described.^[^ ^45^ ^]^ Cells were collected and crosslinked by 1% formaldehyde and further treated with ultrasound. IgG, H3K4me1, H3K4me3, and H3K27ac antibodies were incubated with DNA fragments. The predicted enhancer primer sequences of lncTUBA3FP are listed in Table S3 (Supporting Information).

### Co‐Immunoprecipitation (Co‐IP) Assay

Cells were infected with indicated lentiviruses. Co‐immunoprecipitation was performed by anti‐Flag, anti‐HA, or anti‐TUBORF antibodies. The protein complexes were lysed by IP lysis buffer containing protein inhibitor cocktail, and captured by protein A/G magnetic beads. The secondary antibodies were from IPKine HRP Goat anti‐Mouse or anti‐Rabbit IgG LCS, which could avoid the detection of heavy chains of IgG.

### Immunohistochemistry (IHC)

IHC assay was performed as previously described.^[^ ^46^ ^]^ The cervical cancer tissue microarray chips were fixed with formalin, embedded with paraffin, and immunostained with anti‐TUBORF, anti‐ESCO1 (HUABIO, Wuhan, China), and anti‐IRGQ (Proteintech, Wuhan, China) antibodies. The secondary antibody was HRP‐labeled goat anti‐rabbit antibody (Max Vision, Fuzhou, China). Double‐blinded randomized control design was used to count the percentage of positive cells.

### Soft Agar Colony Formation Assay

Every 1 × 10^4^ cells were plated and cultured in 6‐well culture plates with 10% FBS for two weeks. The numbers and sizes of colonies were photographed under the microscope and counted by the NIH Image J software.

### Immunofluorescence Assay (IFA)

Cells were plated onto glass coverslips in 24‐well plates, fixed with cold acetone for 15 min, permeabilized with 0.25% Triton X‐100, and incubated with 1% BSA in PBST for 30 min. The cells were incubated with antibodies against Flag mouse (1:200) and TUBORF rabbit (1:100) in a humidified chamber overnight at 4 °C, and subsequently incubated with the Alexa Flour 555 anti‐mouse and anti‐rabbit secondary IgG for 1 h. Cellular nuclei were stained with DAPI for 10 min. Images were acquired by a Carl Zeiss laser confocal microscope.

### GSH and GSSG Assay

GSH and GSSG Assay Kit (Beyotime, Jiangsu, China) was used according to the manufacturer's instructions. Every 2 × 10^5^ cells in 6‐well plates were incubated with 10 µm of Erastin for 24 h or 10 mg ground tissue powder frozen in liquid nitrogen washed with PBS, and centrifuged at 13,200 rpm at 4 °C for 10 min. The samples were rapidly freeze‐thawed twice using liquid nitrogen, and 37 °C water and clear lysates were used to determine the amounts of total glutathione (GSH + GSSG). The GSSG content was determined as follows. Briefly, every 100 µL of samples with the total glutathione (GSH + GSSG) content was added with 20 µL of diluted GSH removal auxiliary solution and 4 µL of GSH remove solution at 25 °C for 60 min. The samples were measured at 412 nm with a microplate reader.

### Lipid Peroxidation Detection

Cells were incubated with 1 mL of fresh DMEM added to 5 µm of BODIPY 581/591 C11(Invitrogen) in 6‐well plates for 30 min. Then cells were washed with 1 × PBS for three times, trypsinized, and resuspended in 0.5 mL of PBS for flow cytometry. For in vivo experiments, the tumor tissues were digested with 0.1 mg mL^−1^ collagenase and 1 mg mL^−1^ dispase II, and then crushed through the mesh for single‐cell suspension, finally collected by centrifugation.

### Iron Assay

Cellular ferrous iron (Fe^2+^) was determined using an iron assay kit (Abcam, USA) according to the manufacturer's instructions. Cells were treated with Erastin for 24 h and washed in PBS twice. Every 5 × 10^6^ cells or 100 mg of wet tissue was homogenized in 500 µL of buffer, then centrifuged at 13,200 rpm for 10 min. The supernatant was added with iron probes, followed by incubation for 60 min at 37 °C. The absorbance was measured at 593 nm by a microplate reader.

### Xenograft Studies

Animal studies were performed in accordance with the policies of the Nanjing Medical University Experimental Animal Welfare Ethics Committee. Xenografts were established in 5‐week‐old nude mice (Beijing Vital River Laboratory Animal Technology Corporation) by inoculating 5 × 10^6^ cells mixed in 200 µL PBS. The mice were intraperitoneally injected into the DMSO group and paclitaxel group (15 mg kg^−1^) every 6 days after 7 days of the first subcutaneous injection of SiHa cells. Tumor sizes were measured every 3 days starting from day 7, and tumor volumes were calculated as follows: 1/2 × length × width^2^. After 21 days of treatment, the mice were euthanized and the tumors were removed. A portion of the tumors was immediately detected for ferroptosis indexes.

### Mass Spectrometry

For identification of TUBORF, HeLa cell lysates were separated by SDS‐PAGE and stained with Coomassie blue. The gel bands with ≈10–15 kDa were cut out and analyzed by Mass spectrometry (Nanjing Medical University, Nanjing).

### Statistical Analysis

All experiments were performed at least three times unless otherwise stated. A two‐tailed unpaired Student's *t*‐test of two groups was carried out unless explicitly stated. Statistical analyses were performed using GraphPad Prism 8.0 software, ImageJ software, and the SPSS program. The data were presented as means ± SD. Sample sizes and significance were shown in corresponding figure legends. The comparison results were expressed by ^*^ *p* < 0.05, ^**^ *p* < 0.01, ^***^ *p* < 0.001, and *n.s*. (no statistical significance).

## Conflict of Interest

The authors declare no conflict of interest.

## Author Contributions

X.Q. and J.Z. contributed equally to this work. X.Q., J.Z., X.W., Y.S., Y.C., L.J., M.S., H.Z., T.W., P.W., R.X., Y.Y., and X. D. performed the experiments; X.Q., X.D., X.J., Q.Y., W.L., and C.L. obtained funding for this work; X.Q., Q.Y., W.L., and C.L. drew the pattern diagram; C.W., X.J., Q.Y., W.L., and C.L. conceived the experiments; X.Q., Q.Y., and W.L. writing the original draft; C.W., X.J., Q.Y., W.L., and C.L. review & editing the manuscript.

## Supporting information



Supporting Information

## Data Availability

The data that support the findings of this study are available from the corresponding author upon reasonable request.
