# Supplementary material for: HPV E6/E7‐Induced Acetylation of a Peptide Encoded by a Long Non‐Coding RNA Inhibits Ferroptosis to Promote the Malignancy of Cervical Cancer
Source: Adv Sci (Weinh). 2025 Jan 21;12(10):2414018. doi: 10.1002/advs.202414018 (PMC11905060; doi:10.1002/advs.202414018)
Supplement: Supplementary file 1 — Supporting Information [file ADVS-12-2414018-s001.docx]

Supporting Information

**HPV E6/E7-induced acetylation of a peptide encoded by a long non-coding RNA inhibits ferroptosis to promote the malignancy of cervical cancer**

*Xiaoyu Qi, ^1, 2 †^ Jing Zhou, ^2 †^ Xinyue Wang, ^2^ Yan Shen, ^2^ Yuxun Cao, ^2^ Liangzi Jiang, ^2^ Miaomiao Shen, ^3^ Haoran Zhang, ^2^ Tianjiao Wang, ^2^ Pengjun Wei, ^2^ Ruoqi Xu, ^2^ Yue Yang, ^2^ Xiangya Ding, ^1, 2^ Cong Wang, ^3^ * Xuemei Jia, ^1^ * Qin Yan, ^2, 5^ * Wan Li, ^1, 2, 4, 5^ * and Chun Lu ^1, 2, 4, 5^ **

This file includes:

Figure S1 to S13

Table S1 to Table S3

Supplementary figures


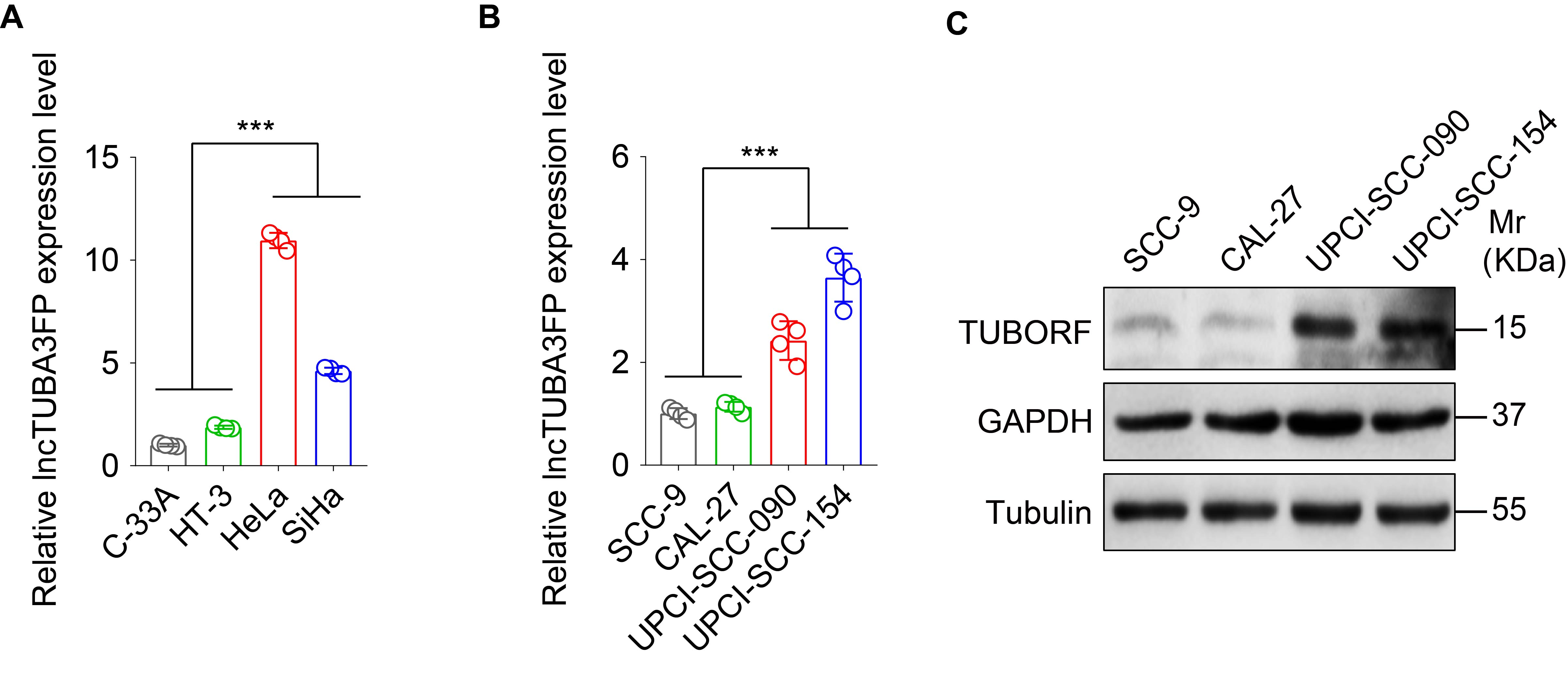


**Figure S1. The expression level of lncTUBA3FP and TUBORF in HPV-negative and HPV-positive cell lines.**

(**A**). RT-qPCR analysis of lncTUBA3FP level in HPV-negative cervical cancer cell lines (C-33A and HT-3) and HPV-positive cervical cancer cell lines (HeLa and SiHa) (*n*=4).

(**B**). RT-qPCR analysis of lncTUBA3FP level in HPV-negative human tongue squamous cell lines (SCC-9 and CAL-27) and HPV-positive human tongue squamous cell lines (UPCI-SCC-090 and UPCI-SCC-154) (*n*=4).

(**C**). Western blot analysis of the TUBORF peptide level in HPV-negative human tongue squamous cell lines (SCC-9 and CAL-27) and HPV-positive human tongue squamous cell lines (UPCI-SCC-090 and UPCI-SCC-154).


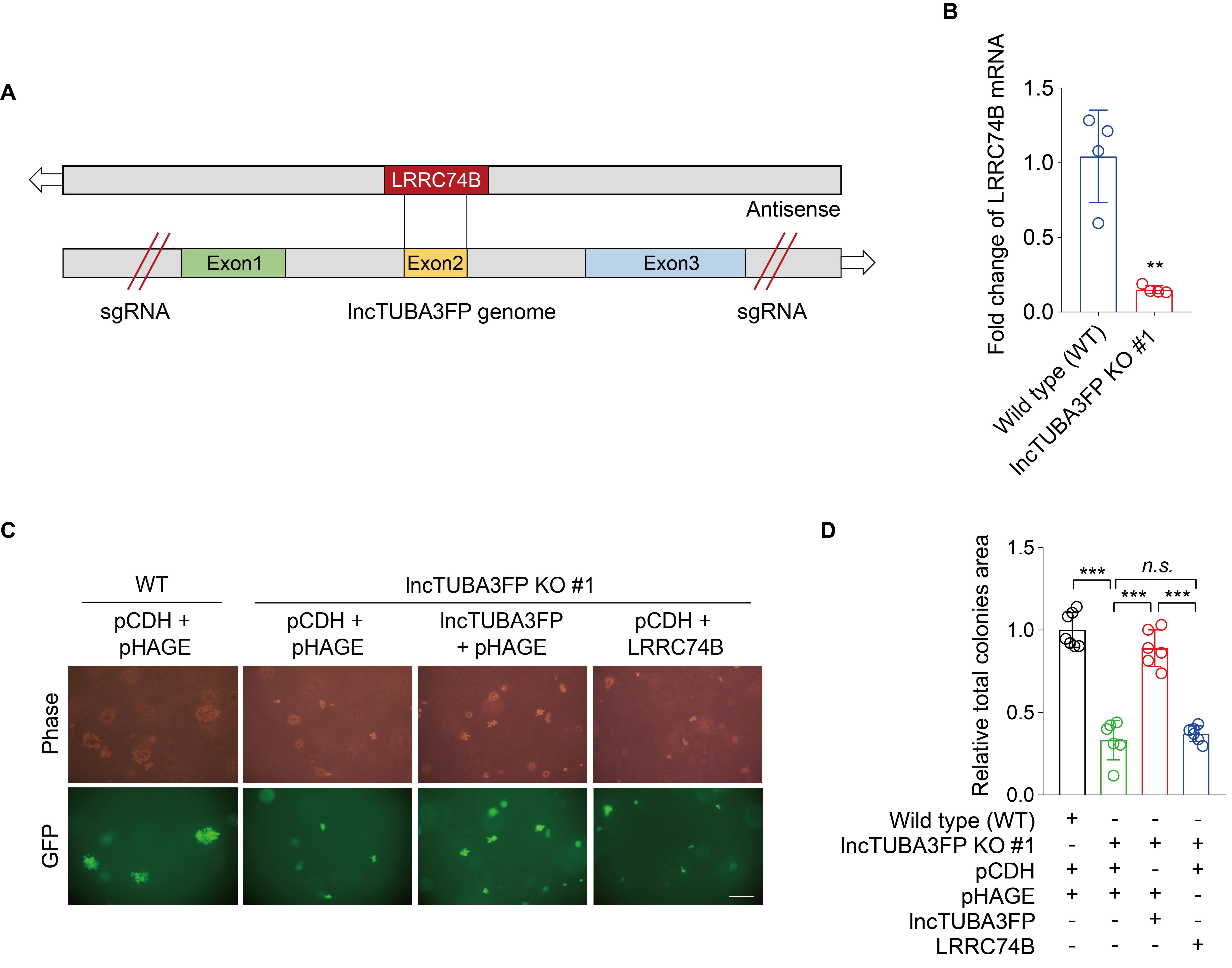


**Figure S2. TUBORF peptide, not LRRC74B generated from lncTUBA3FP, inhibits ferroptosis and promotes the malignant proliferation of cervical cancer cells.**

(**A**). Pattern diagram of the location of *LRRC74B* gene in lncTUBA3FP genome.

(**B**). RT-qPCR analysis of LRRC74B mRNA level in lncTUBA3FP knockout HeLa cells (**lncTUBA3FP KO #1**) or the wild type cells (**WT**) (*n*=4).

(**C**). Soft agar colony formation assay was performed with lncTUBA3FP knockout HeLa cells (**lncTUBA3FP KO #1**) or the wild type cells (**WT**) transduced with lentiviral lncTUBA3FP or LRRC74B, respectively. The representative images were photographed at two weeks after cells seeding. Magnification, ×100. Scar bars, 40 μm.

(**D**). Results were quantified in (**C**) (*n*=6-7).

Data were presented with mean ± SD. ** *P* < 0.01, and *** *P* < 0.001, Student's *t*-test. *n.s.*, not significant.


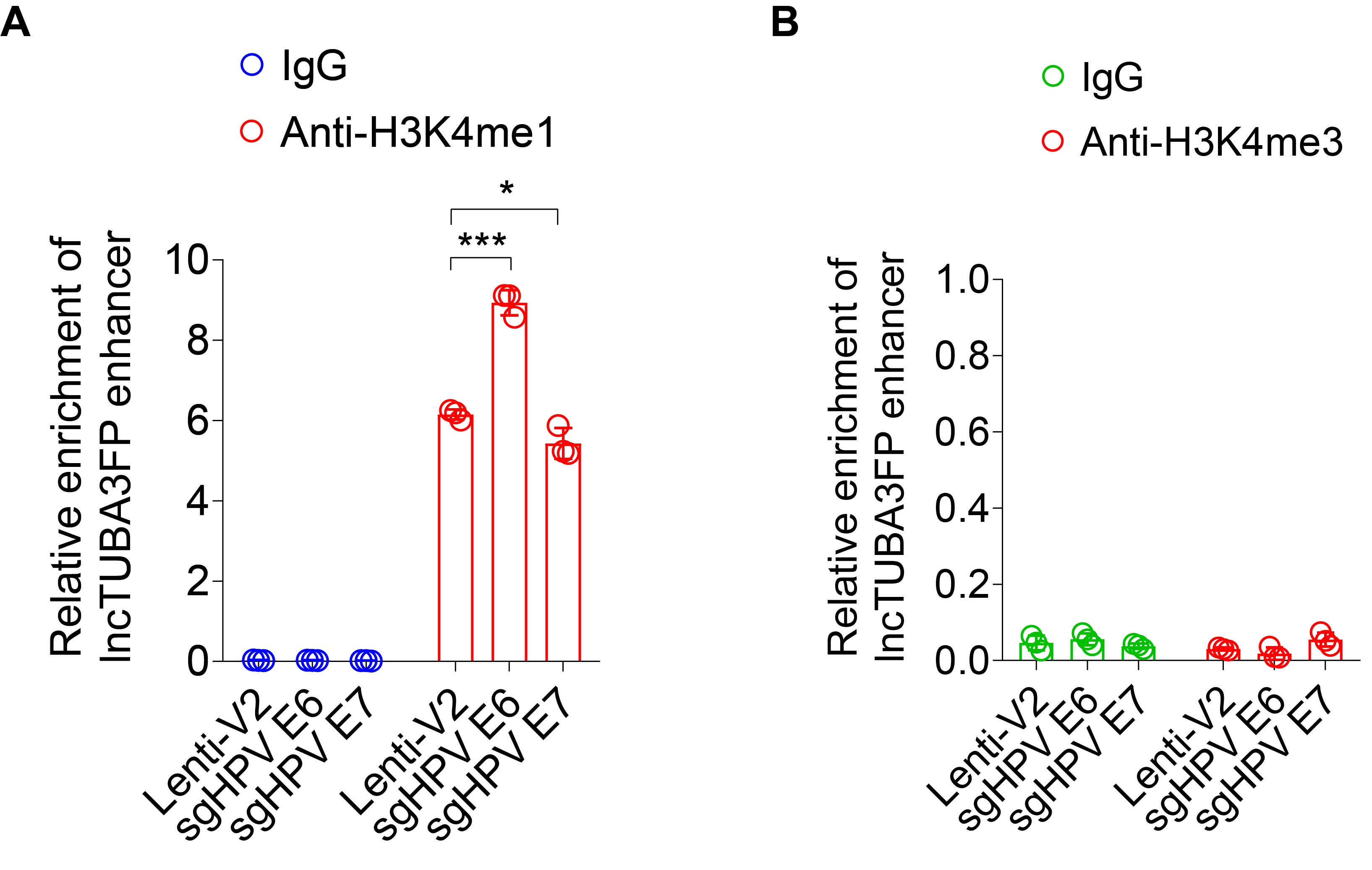


**Figure S3. Either H3K4me1 or H3K4me3 of lncTUBA3FP enhancer is not regulated by HPV E6 or E7 in cervical cancer cells.**

(**A**). ChIP-qPCR analysis of the H3K4me1 level in lncTUBA3FP enhancer in E6- or E7-knockdown HeLa cells (**sgHPV E6**, **sgHPV E7**) or the control (Lenti-V2) (*n*=3).

(**B**). ChIP-qPCR analysis of the H3K4me3 level in lncTUBA3FP enhancer in E6- or E7-knockdown HeLa cells (**sgHPV E6**, **sgHPV E7**) or the control (Lenti-V2) (*n*=3).

Data were presented with mean ± SD. * *P* < 0.05, and *** *P* < 0.001, Student's *t*-test.


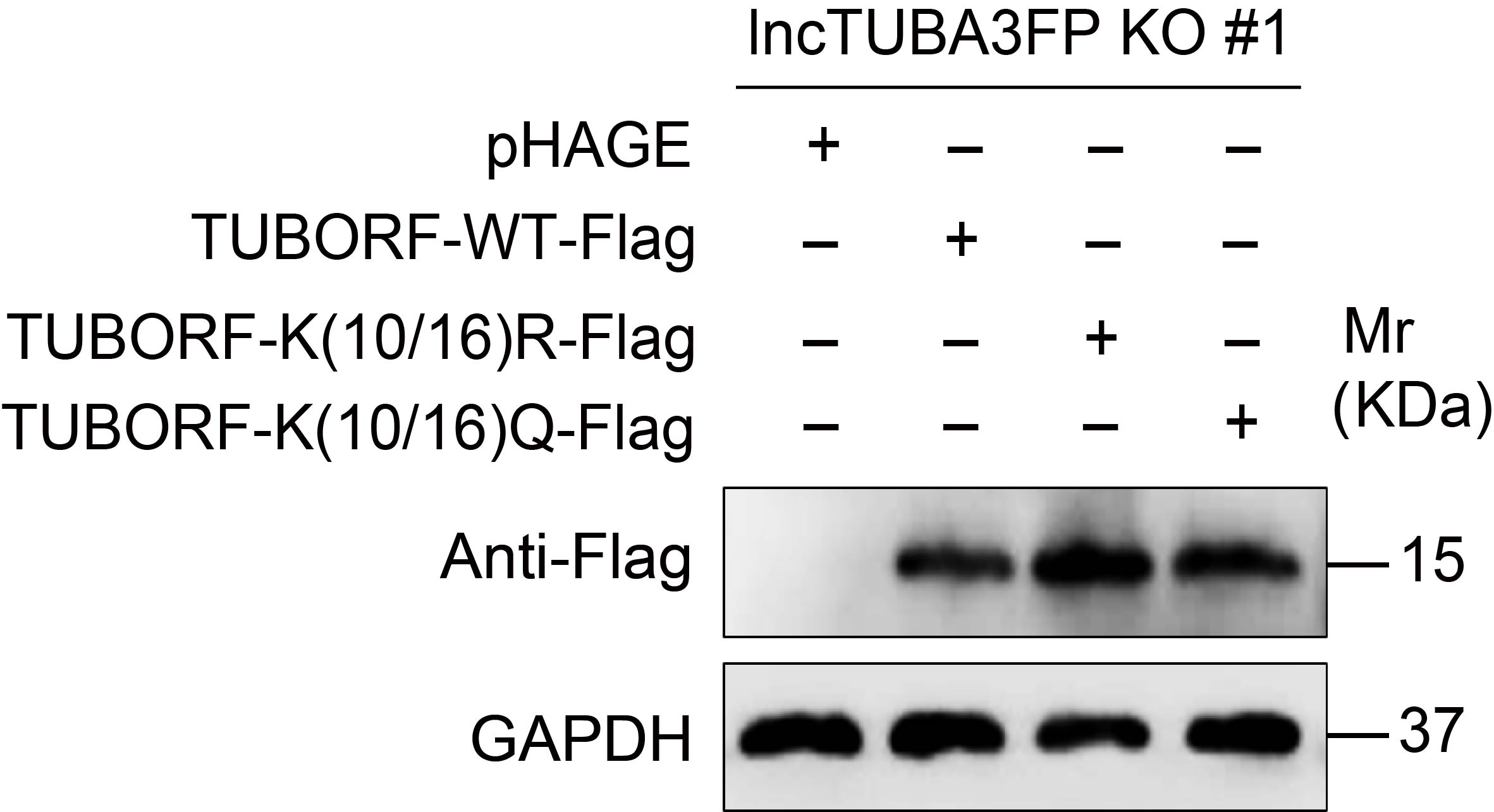


**Figure S4. Overexpression of wild type TUBORF, K(10/16)R and K(10/16)Q mutant TUBORF in lncTUBA3FP knockout cervical cancer cells.**

Western blotting was performed to examine TUBORF expression in lncTUBA3FP knockout HeLa cells (**lncTUBA3FP KO #1**) transduced with lentiviral TUBORF-WT, TUBORF-K(10/16)R, TUBORF-K(10/16)Q or control lentivirus pHAGE with anti-Flag antibody.


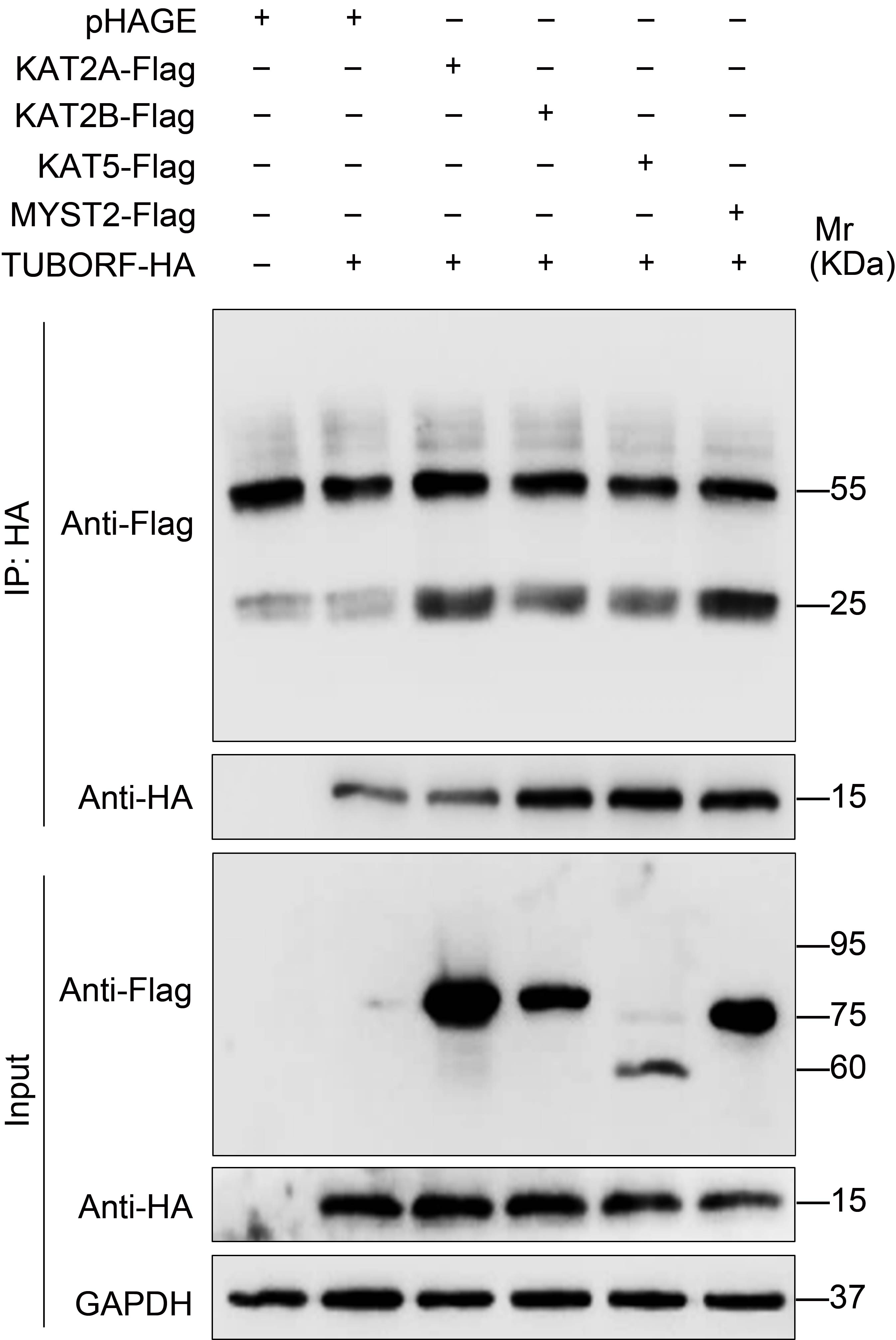


**Figure S5. TUBORF peptide does not interact with the indicated acetyltransferases.**

Cells were co-transduced by TUBORF-HA (**TUBORF-HA**) and KAT2A-Flag (**KAT2A-Flag**), KAT2B-Flag (**KAT2B-Flag**), KAT5-Flag (**KAT5-Flag**) or MYST2-Flag (**MYST2-Flag**), respectively. Immunoprecipitation assay was performed to detect the interaction between the acyltransferases and TUBORF peptide with the anti-HA antibody.


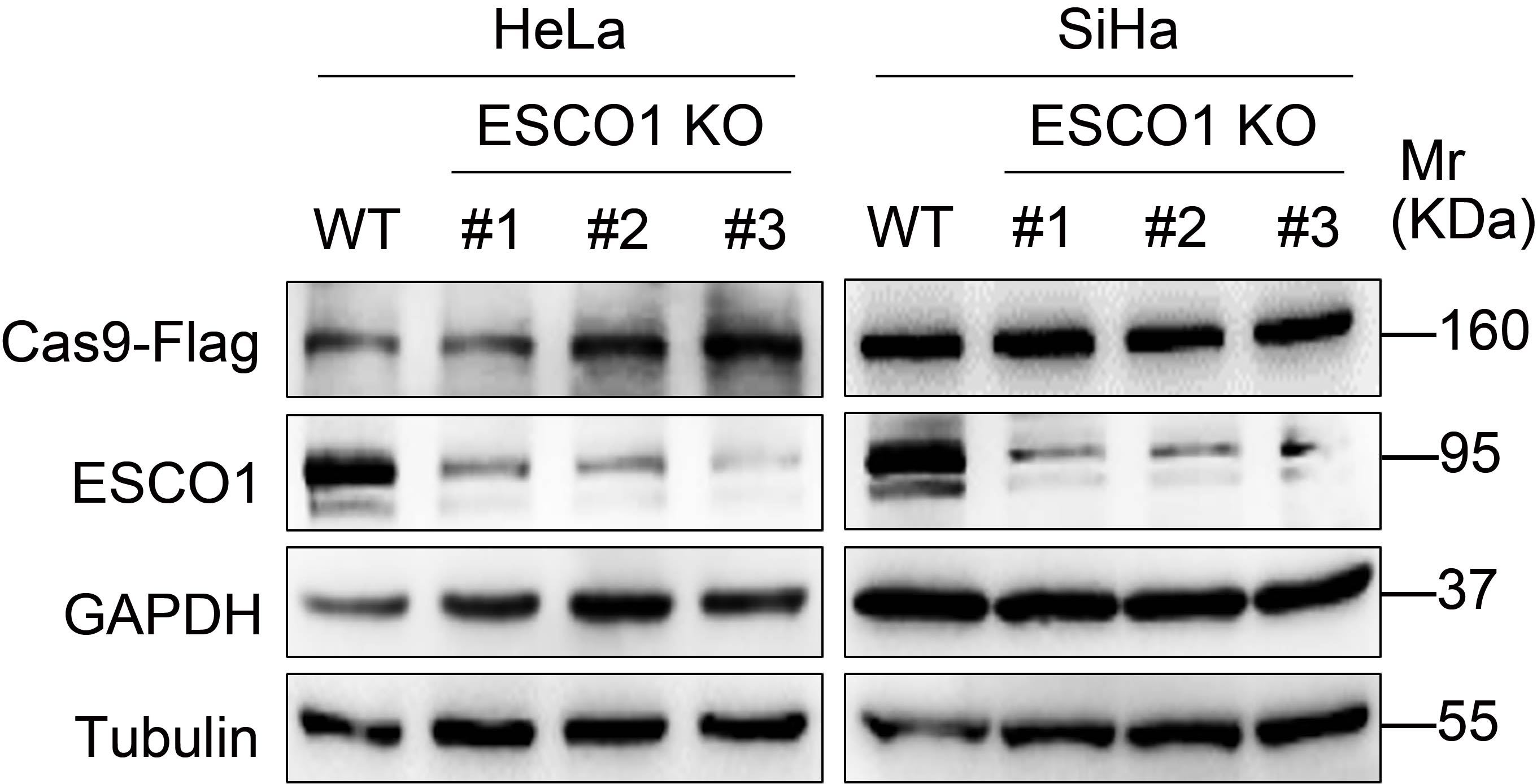


**Figure S6. Knockout efficiency of ESCO1 in cervical cancer cells.**

Western blotting analysis of ESCO1 expression in ESCO1 knockout cervical cancer cells using CRISPR/Cas9 method. Three monoclonal cells of cervical cancer with ESCO1 knockout were screened and identified (**ESCO1 KO #1, KO #2, and KO #3**).


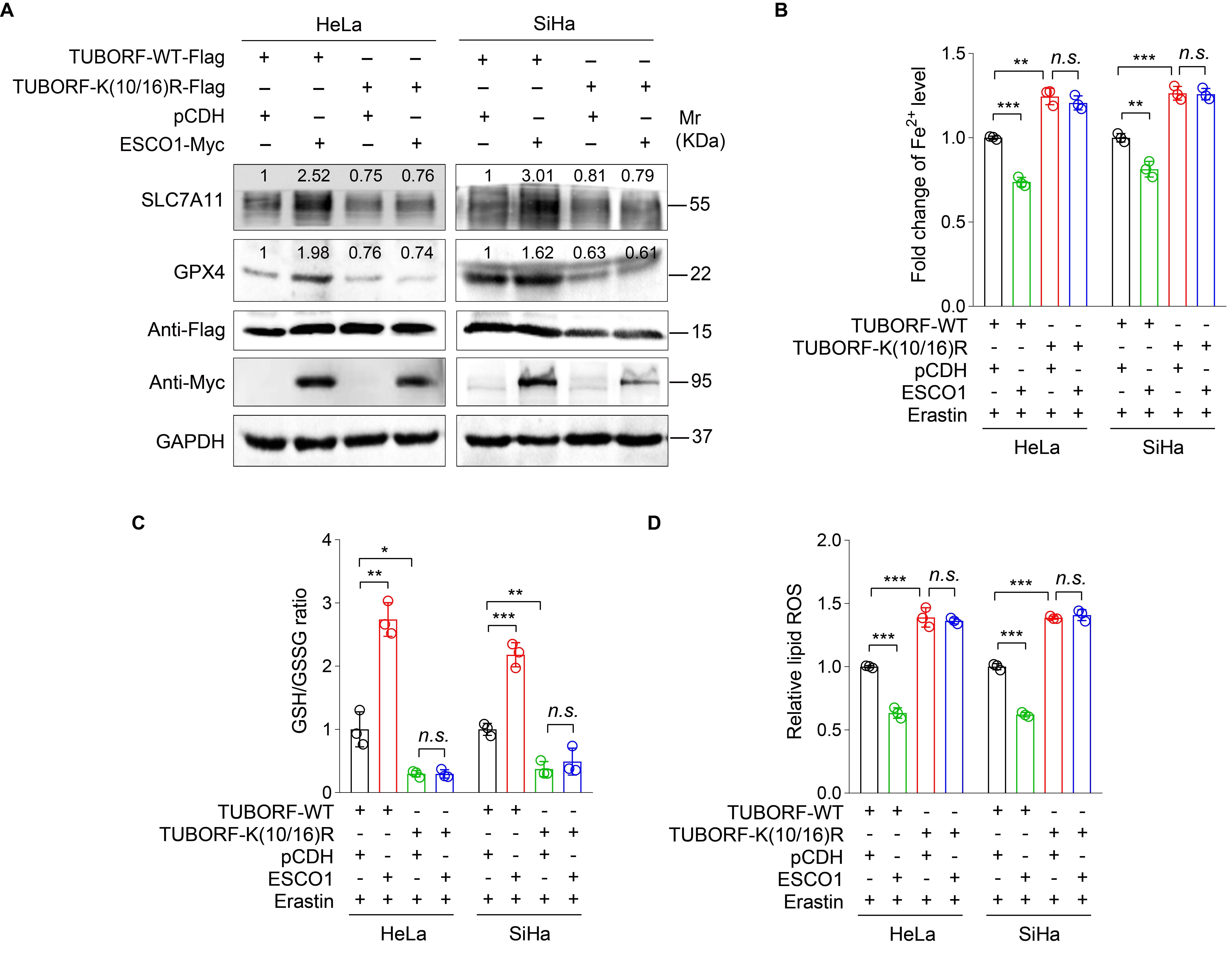


**Figure S7. The function of ESCO1 on ferroptosis was achieved by acetylated TUBORF.**

**(A).** Cells transduced with TUBORF-WT (**TUBORF-WT-Flag**) and TUBORF-K(10/16)R (**TUBORF-K(10/16)R-Flag**) were overexpressed with ESCO1 (**ESCO1-Myc**) and pCDH (**pCDH**). Cells were then treated with 5 µM Erastin for 24 h and subjected to Western blotting analysis for detection of SLC7A11 and GPX4 expression.

**(B).** Cells treated as in (**A**) were employed to examine Fe^2+^ levels (*n*=3).

**(C).** Cells treated as in (**A**) were used to measure GSH/GSSG levels (*n*=3).

**(D).** Cells treated as in (**A**) were used to examine lipid ROS levels (*n*=3).

Data were presented with mean ± SD. * *P* < 0.05, ** *P* < 0.01, and *** *P* < 0.001, Student's *t*-test. *n.s.*, not significant.


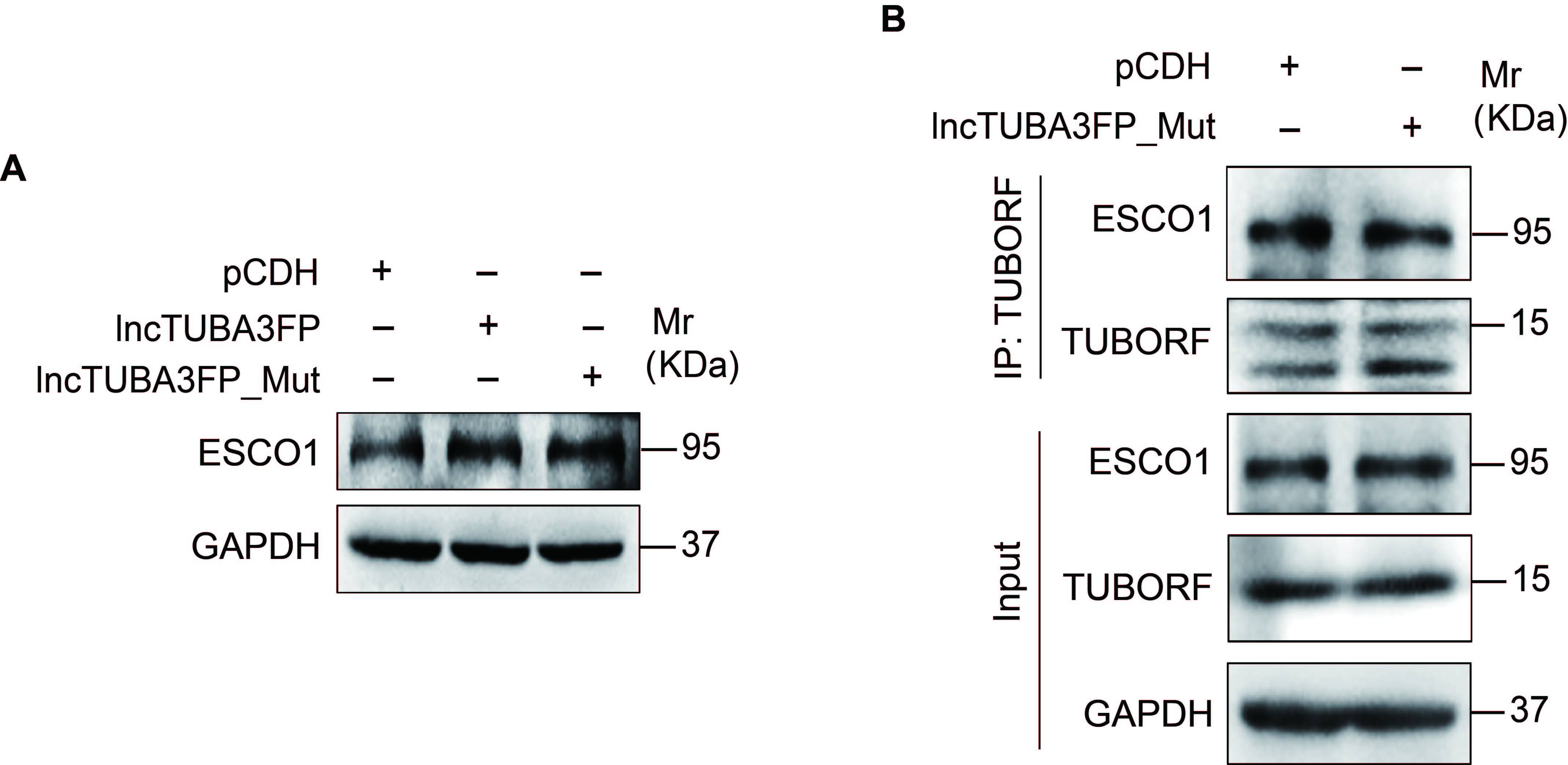


**Figure S8. LncTUBA3FP does not influence the abundance of ESCO1 nor affect its binding affinity for TUBORF.**

**(A).** Western blotting analysis of ESCO1 expression in HeLa cells transduced by 2 MOI lentiviral pCDH (**pCDH**), lncTUBA3FP (**lncTUBA3FP**), and lncTUBA3FP_Mut (**lncTUBA3FP_Mut**).

**(B).** IP assay analysis of the interaction between ESCO1 and TUBORF in HeLa cells transduced by 2 MOI lentiviral pCDH (**pCDH**) and lncTUBA3FP_Mut (**lncTUBA3FP_Mut**).


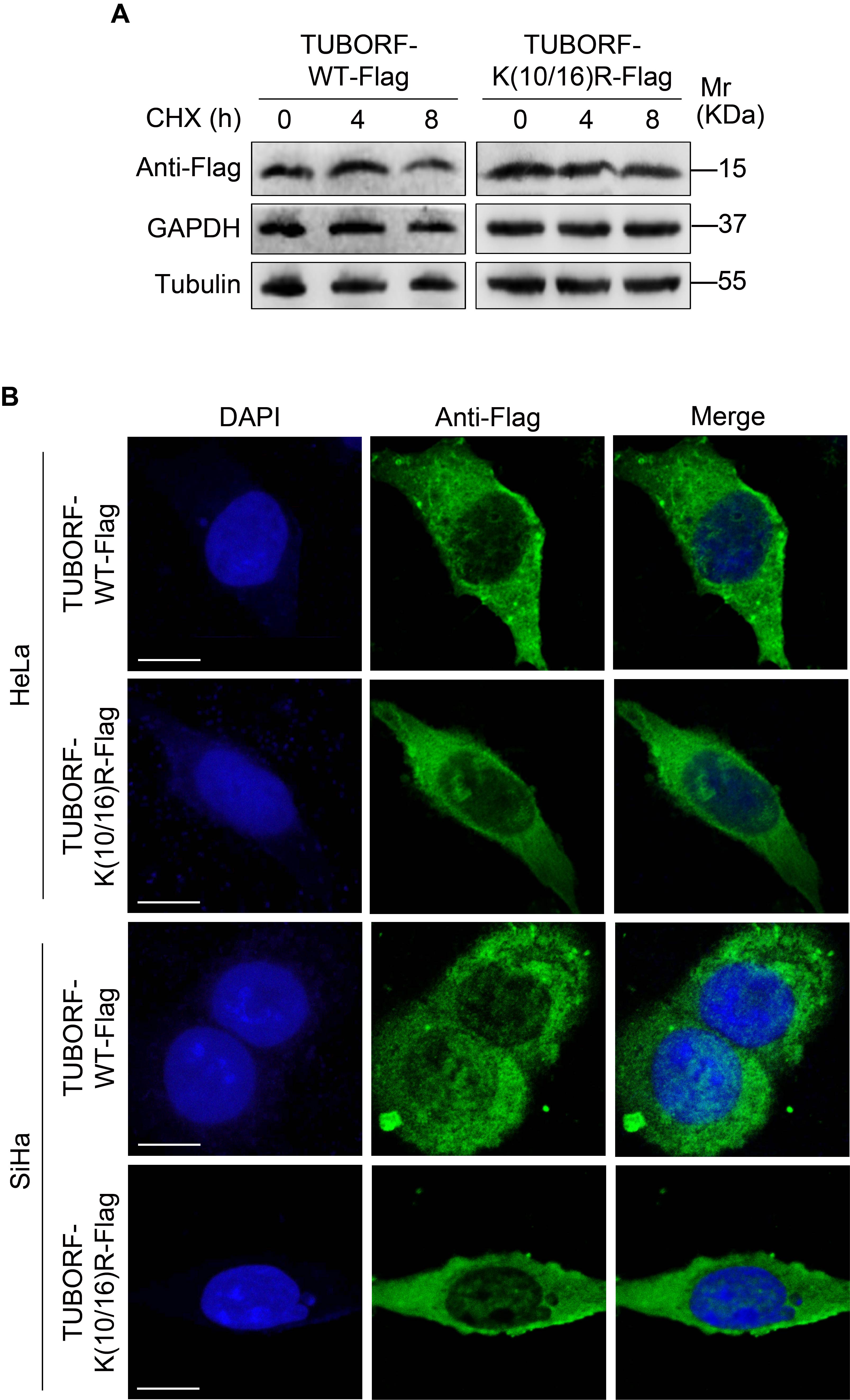


**Figure S9. Acetylation modification does not affect the stability and localization of TUBORF in cervical cancer cells.**

**(A).** HeLa cells transduced with wild-type TUBORF (**TUBORF-WT-Flag**) or K10/16R mutant TUBORF (**TUBORF-K(10/16)R**) were treated with CHX (20 μg/mL) for 0, 4 and 8 h, respectively. Western blotting analysis was performed to examine TUBORF expression.

**(B).** Immunofluorescence staining (IFA) analysis of TUBORF peptide localization in HeLa and SiHa cells transduced with wild-type TUBORF (**TUBORF-WT-Flag**) or K10/16R mutant TUBORF (**TUBORF-K(10/16)R**) .


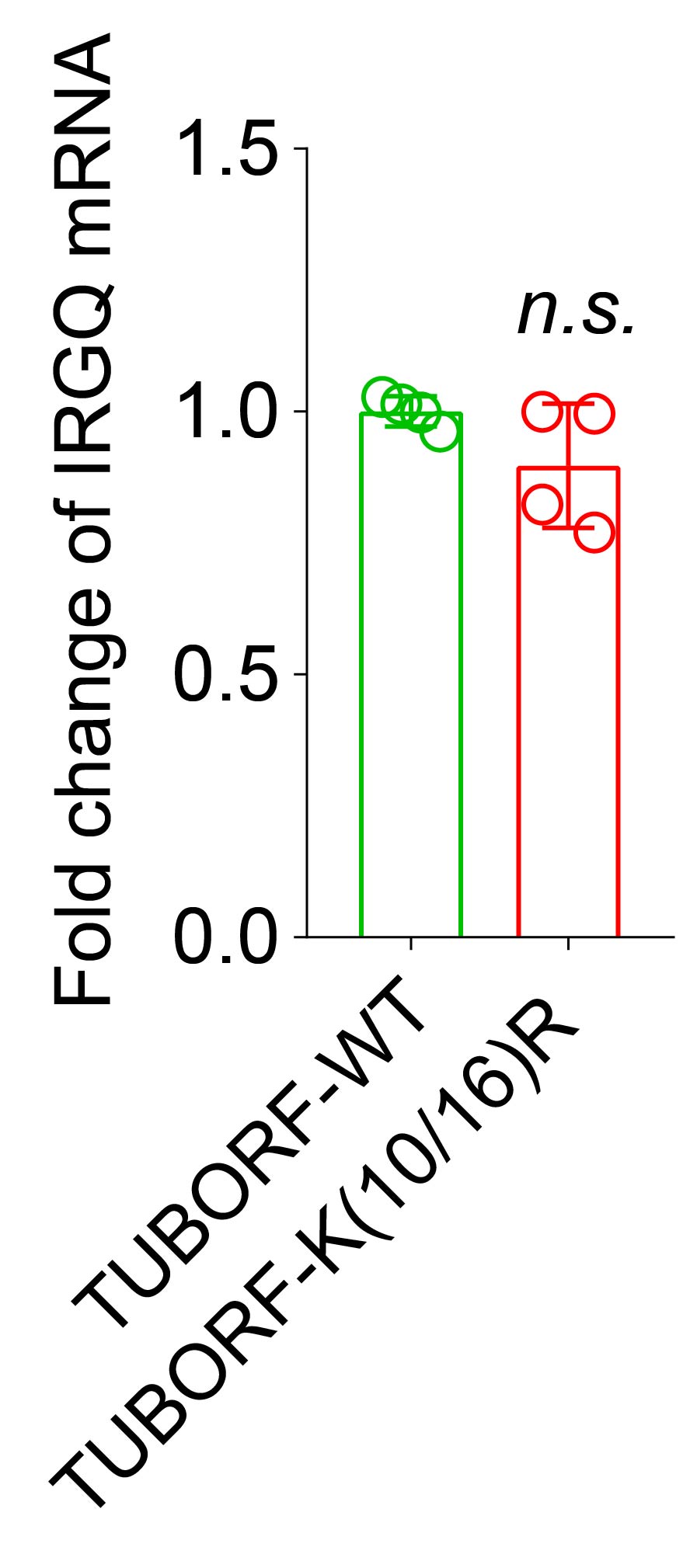


**Figure S10. TUBORF acetylation has no effect on IRGQ mRNA level.**

qRT-PCR analysis of IRGQ mRNA level in HeLa cells transduced with lentiviral TUBORF-WT (**TUBORF-WT**) or TUBORF-K10/16R (**TUBORF-K(10/16)R**) (*n*=4). *n.s.*, not significant.


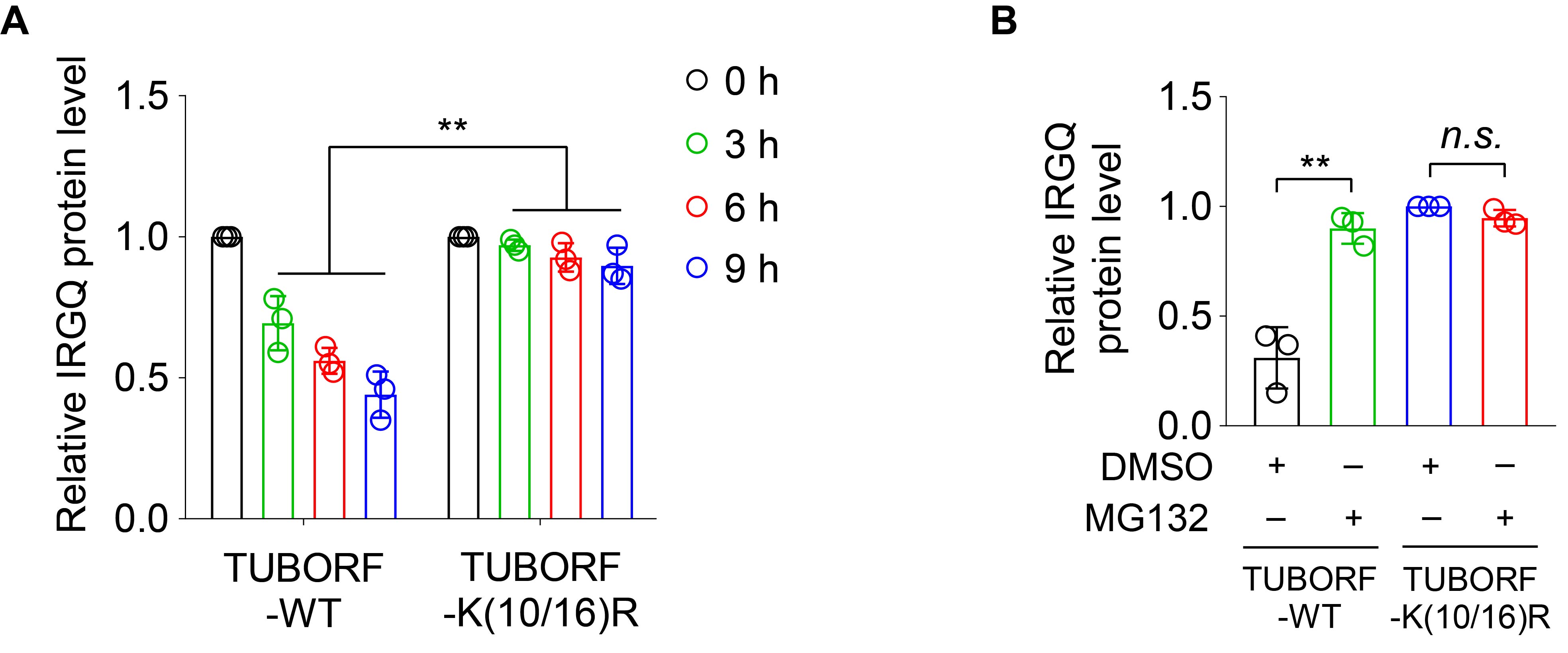


**Figure S11. Acetylated TUBORF promotes IRGQ protein degradation through the proteasome pathway.**

**(A).** Results were quantified in **Figure 7F** (*n*=3).

**(B).** Results were quantified in **Figure 7G** (*n*=3).

Data were presented with mean ± SD. ** *P* < 0.01, Student's *t*-test. *n.s.*, not significant.


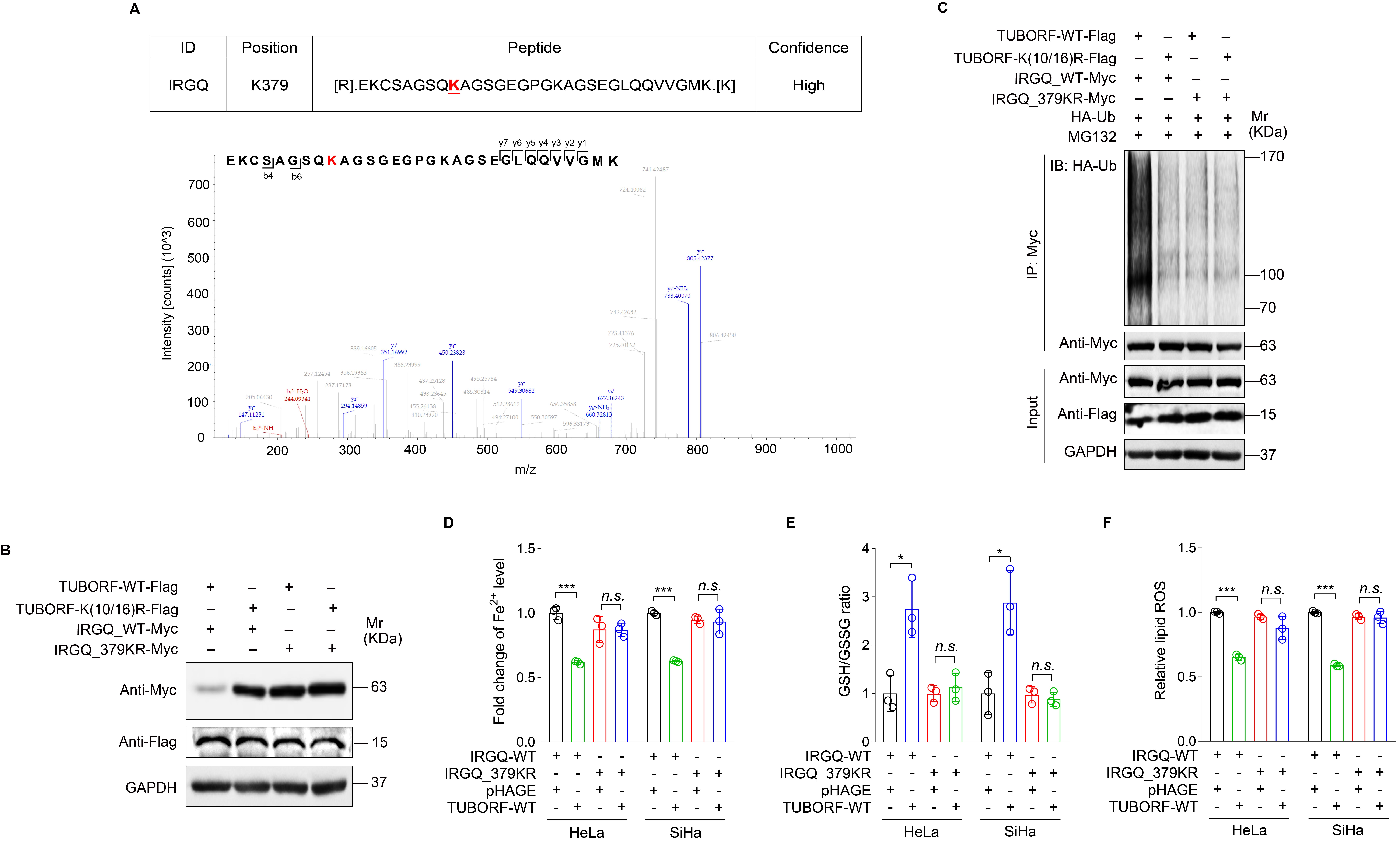


**Figure S12. Acetylated TUBORF promotes ferroptosis by enhancing IRGQ ubiquitination modification.**

**(A).** The possible ubiquitination site of IRGQ was predicted by immunoprecipitation (IP)-mass spectrometry.

**(B).** Lentiviral TUBORF-WT (**TUBORF-WT**) or TUBORF-K10/16R (**TUBORF-K(10/16)R**) transduced HeLa cells were infected with wild type IRGQ (**IRGQ_WT-Myc**) or mutant IRGQ (**IRGQ_379KR-Myc**). Western blotting was performed to examine IRGQ expression level with anti-Myc antibody.

**(C).** HA-Ub-overexpressing HeLa cells were infected with wild type IRGQ (**IRGQ_WT-Myc**) or mutant IRGQ (**IRGQ_379KR-Myc**) and further transduced with lentiviral TUBORF-WT or TUBORF-K(10/16)R. Cells were then treated with MG132 (10 µM) for 6 h and subjected to immunoprecipitation assay (IP) for detection of IRGQ ubiquitination with anti-Myc antibody.

**(D).** Cells transduced with IRGQ-WT (**IRGQ-WT-Myc**) and IRGQ_379KR (**IRGQ_379KR-Myc**) were overexpressed with TUBORF (**TUBORF-Flag**) and pHAGE (**pHAGE**). Cells were treated with 5 µM Erastin for 24 h and then were employed to examine Fe^2+^ levels (*n*=3).

**(E).** Cells treated as in (**D**) were used to measure GSH/GSSG levels (*n*=3).

**(F).** Cells treated as in (**D**) were used to examine lipid ROS levels (*n*=3).

Data were presented with mean ± SD. * *P* < 0.05 and *** *P* < 0.001, Student's *t*-test. *n.s.*, not significant.


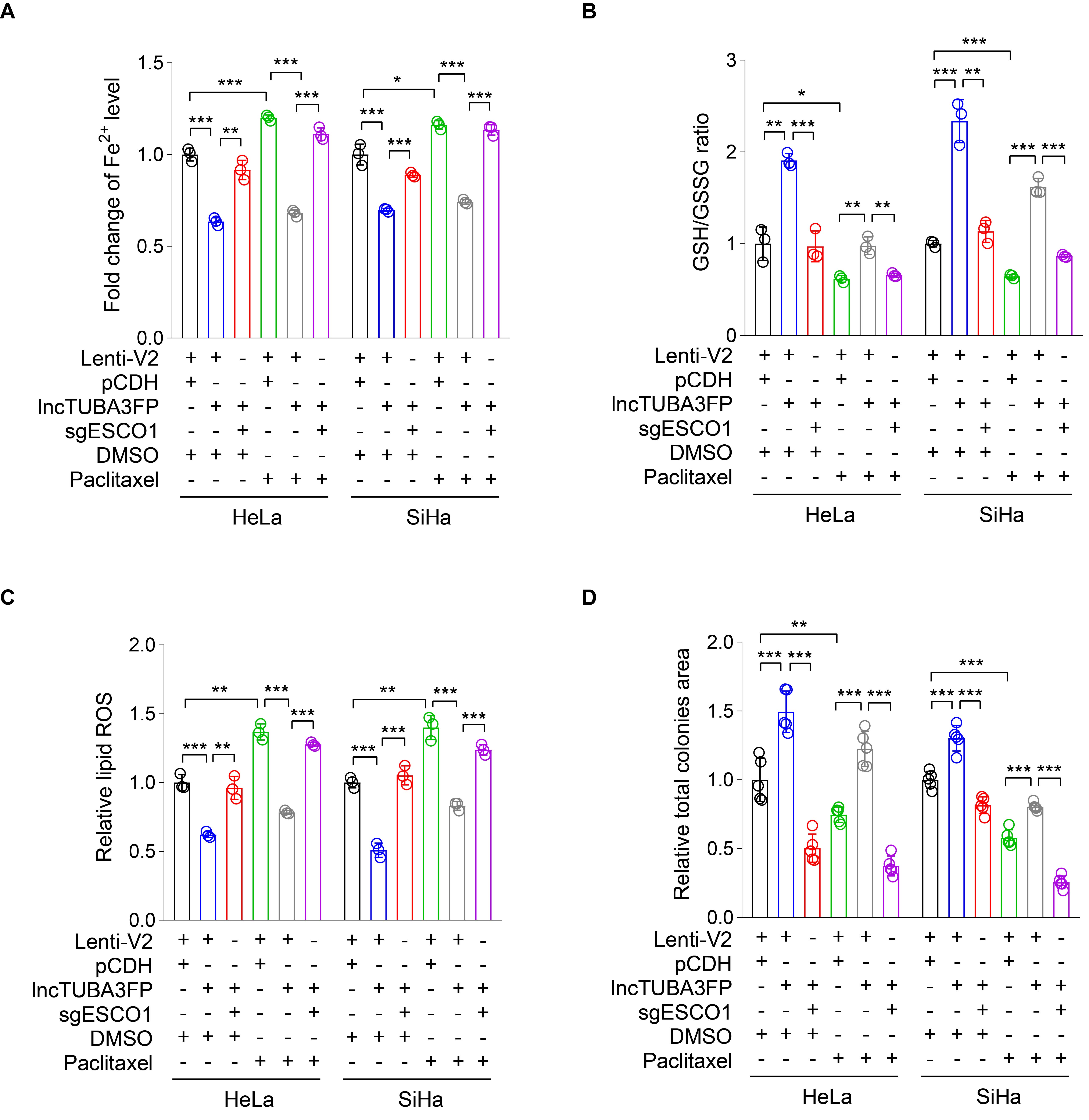


**Figure S13. Knockdown of ESCO1 rescues lncTUBA3FP-mediated ferroptosis inhibition.**

**(A).** HeLa and SiHa cells overexpressed with lncTUBA3FP or pCDH and subsequently silenced for ESCO1 (**sgESCO1**) were treated with DMSO or 10 μM paclitaxel for 24 h, followed by a treatment with 5 µM Erastin for 24 h, and then employed to examine Fe^2+^ levels (*n*=3).

**(B).** Cells treated as in (**A**) were used to measure GSH/GSSG levels (*n*=3).

**(C).** Cells treated as in (**A**) were used to examine lipid ROS levels (*n*=3).

**(D).** Soft agar colony formation assay of cells treated as in (**A**) (*n*=5).

Data were presented with mean ± SD. * *P* < 0.05, ** *P* < 0.01, and *** *P* < 0.001.

**Table S1. Correlation between TUBORF expression and clinicopathological parameters in 287 cervical cancer patients.**

| **Clinicopathological Parameters** | **TUBORF expression, n (%)** | | ***P* value** |
| --- | --- | --- | --- |
|  | **High ≥2** | **Low 0-1** |  |
| **Age** |  |  |  |
| ≤45 | 99 (80.5) | 24 (19.5) | 0.213 |
| >45 | 134 (81.7) | 30 (18.3) |  |
| **Tumor size** |  |  |  |
| ≤4 cm | 186 (81.9) | 41 (18.1) | 0.199 |
| >4 cm | 47 (78.3) | 13 (21.7) |  |
| **Depth of cervical stromal invasion** |  |  |  |
| ≤1/2 full cervical layer | 64 (85.3) | 11 (14.7) | 0.890 |
| >1/2 full cervical layer | 169 (79.7) | 43 (20.3) |  |
| **Lymph node metastasis** |  |  |  |
| No | 192 (80.7) | 46 (19.3) | 0.883 |
| Yes | 41 (83.7) | 8 (16.3) |  |
| **Lymphovascular invasion** |  |  |  |
| No | 107 (82.9) | 22 (17.1) | 0.842 |
| Yes | 126 (79.7) | 32 (20.3) |  |
| **Histopathologic stage** |  |  |  |
| ≤Ⅱ | 98 (83.1) | 20 (16.9) | 0.592 |
| >Ⅱ | 135 (79.9) | 34 (20.1) |  |
| **FIGO stage** |  |  |  |
| Ⅰ | 136 (81.0) | 32 (19.0) | 0.874 |
| Ⅱ | 56 (80.0) | 14 (20.0) |  |
| Ⅲ | 41 (83.7) | 8 (16.3) |  |
| **p16 status** |  |  |  |
| Positive | 219 (77.4) | 64 (22.6) | 0.110 |
| Negative | 2 (50.0) | 2 (50.0) |  |

**0:** No staining was observed, or ≤10% of infiltrating cancer cells demonstrated incomplete and faint staining; **1:** >10% of infiltrating cancer cells exhibited incomplete and faint staining; **2:** Either ≤10% infiltrating cancer cells showed strong and complete staining, or >10% infiltrating cancer cells presented weak-medium strength staining; **≥2:** >10% of infiltrating cancer cells exhibited strong and complete staining. **Table S2. The sequences of sgRNAs**

| **Target** | **Primer** |
| --- | --- |
| lncTUBA3FP-sgRNA-1 | F: 5'-CACCG CCGTGGCAGACGCCAGTCGA-3'  R: 5'-AAACTCGACTGGCGTCTGCCACGGC-3' |
| lncTUBA3FP-sgRNA-2 | F: 5'-CACCGTATGACAGATAAGAACATCG-3'  R: 5'-AAACCGATGTTCTTATCTGTCATAC-3' |
| lncTUBA3FP-sgRNA-3 | F: 5'-CACCGCGGAGCCACTCCTCAGCCAG-3'  R: 5'-AAACCTGGCTGAGGAGTGGCTCCGC-3' |
| lncTUBA3FP-sgRNA-4 | F: 5'- CACCGCAGAGGAACAGTGAGCATGC-3'  R: 5'-AAACGCATGCTCACTGTTCCTCTGC-3' |
| lncTUBA3FP-sgRNA-5 | F: 5'-CACCGAGATCTGTGCATGGGACTGC-3'  R: 5'-AAACGCAGTCCCATGCACAGATCTC-3' |
| lncTUBA3FP-sgRNA-6 | F: 5'-CACCGTCATTGAGACTTATAAATGT-3'  R: 5'-AAACACATTTATAAGTCTCAATGAC-3' |
| ESCO1-sgRNA1 | F: 5‘-AAGGCTACAACAATTAACAG-3’  R: 5’-CTGTTAATTGTTGTAGCCTT-3’ |
| ESCO1-sgRNA2 | F: 5‘-CAGCCAAGAAGAACTCCAAC-3’  R: 5’-GTTGGAGTTCTTCTTGGCTG-3’ |
| ESCO1-sgRNA3 | F: 5‘-AAAGAATGAGATGAAGAAGT-3’  R: 5’-ACTTCTTCATCTCATTCTTT-3’ |
| HPV18 E6-sgRNA1 | F: 5‘-GCGCTTTGAGGATCCAACA-3’  R: 5’-TGTTGGATCCTCAAAGCGC-3’ |
| HPV18 E6-sgRNA2 | F: 5‘-AAGCTACCTGATCTGTGCA-3’  R: 5’-TGCACAGATCAGGTAGCTT-3’ |
| HPV18 E7-sgRNA1 | F: 5‘-GAGCAATTAAGCGACTCAG-3’  R: 5’-CTGAGTCGCTTAATTGCTC-3’ |
| HPV18 E7-sgRNA2 | F: 5‘-GAAGAAAACGATGAAATAGA-3’  R: 5’-TCTATTTCATCGTTTTCTTC-3’ |

F: forward; R: reverse

**Table S3. The sequences of primers for ChIP**

| **Target** | **Application** | **Primer** |
| --- | --- | --- |
| lncTUBA3FP | H3K27ac-qPCR | F: 5'-CATCATAGGACCTGCCACCTG-3'  R: 5'-TGTGCTTACTCTTGGTGAACCC-3' |
| lncTUBA3FP | H3K4Me1-qPCR | F: 5'-GCCCACAAAGATGAAAGCTG-3'  R: 5'-AAGCTGGAATATCACCTCCC-3' |
| lncTUBA3FP | H3K4Me3-qPCR | F: 5'-TCCAGCCAAATTTCAGAACGG-3'  R: 5'-TGTGCTTACTCTTGGTGAACCC-3' |

F: forward; R: reverse
